# Multiplatform Benchtop NMR Interlaboratory Study of Model Liquid Dosage Forms of Pharmaceutical Products

**DOI:** 10.1021/acs.analchem.5c05487

**Published:** 2026-01-05

**Authors:** Katharine T. Briggs, Frank Delaglio, Marc B. Taraban, Robert G. Brinson, Luke Arbogast, Brendan Lichtenthal, Matteo Pennestri, Robert Espina, Hector Robert, Juan F. Araneda, Paul Hui, Susanne D. Riegel, Kevin Nott, Leonid Grunin, Innokenty Nikolaev, Thomas Reininger, Y. Bruce Yu, John P. Marino

**Affiliations:** † Institute for Bioscience and Biotechnology Research, 12265 University of Maryland, Baltimore, Rockville, Maryland 20850, United States; ‡ Institute for Bioscience and Biotechnology Research, 10833 National Institute of Standards and Technology, Rockville, Maryland 20850, United States; § Bruker BioSpin Corp., 15 Fortune Drive, Billerica, Massachusetts 01821, United States; ∥ Bruker BioSpin Corp., Banner Lane, Coventry CV4 9GH, U.K.; ⊥ 383888 Magritek, Inc., 103 Great Valley Parkway, Malvern, Pennsylvania 19355, United States; # 434748 Nanalysis Corp., 1-4600 5 Street Northeast, Calgary, Alberta T2E 7C3, Canada; ¶ Oxford Instruments Magnetic Resonance, Tubney Woods, Abingdon OX13 5QX, U.K.; ∇ Resonance Systems, GmbH Seestrasse 28, D-73230 Kirchheim/Teck, Germany

## Abstract

Low-field NMR relaxometry uses water proton (^1^H_2_O) relaxation (*w*NMR) as a powerful and flexible tool to reveal deviations in critical quality attributes of liquid pharmaceuticals such as concentration or, in the case of proteins, aggregation. Measurements are fast and nondestructive, and many benchtop instruments can make measurements noninvasively, with an intact drug vial, prefilled syringe, or pen inserted directly into the probe. Because of the diversity of low-field instrument configurations and operating temperatures, the varying field strengths available, and the many possible protocols, pulse sequences, and parameters for conducting relaxation measurements, we sought to test the reproducibility of *w*NMR across platforms, and to identify the details of applying *w*NMR that impact reproducibility. To accomplish this, we piloted an interlaboratory study, in collaboration with benchtop NMR instrument vendors, using model liquid dosage forms of pharmaceutical products in sealed vials. This study demonstrates that when suitable measurement protocols are employed, *w*NMR measurements from all instruments can track the concentrations of diverse samples with high linearity. Furthermore, *w*NMR measurements were capable of detecting intentionally included outlier samples. This study revealed various operating procedures and variables that contributed more to measurement variance than any hardware- or instrument-specific differences. Based on these results, we offer initial considerations for harmonizing measurement protocols to enable reliable *w*NMR analysis of pharmaceuticals.

## Graphical abstract

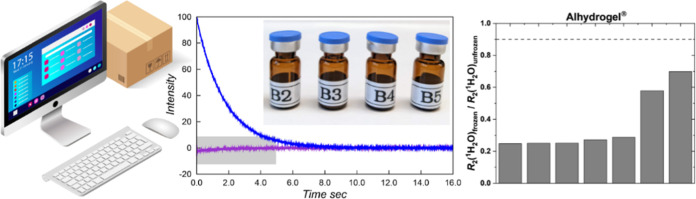

## Introduction

Benchtop NMR has proven to be a useful tool for R&D and quality control in the food and oil industries.^1,2^ In the pharmaceutical and biotech industries, high-field NMR spectroscopy, which requires cryogenic superconducting magnets, is routinely used as an analytical tool in R&D^3^ and is gaining use as a method for higher order structure measurement^4^ but is not well-suited for use in a manufacturing environment. Benchtop NMR is more compatible with in-line and online analytics in manufacturing, but its potential has yet to be fully realized. Pharmaceutical companies work to produce safe and effective drug products and vaccines, often with an aggressive timeline for production and scale-up for manufacture, which brings in to focus the need for quality assurance technologies.^5,6^ Implementation of benchtop NMR quality control measurements could save time, money, and lives if implemented at various checkpoints in the pharmaceutical pipeline.^7^

Groundwork in the application of benchtop NMR indicates that the transverse relaxation rate of the water proton signal, *R*_2_(^1^H_2_O), also called water NMR (*w*NMR), can, quite usefully, detect changes in drug concentration,^8^ protein aggregation content,^9^ exposure of vaccines to freeze/thaw cycles,^10^ and can serve as a contact-free, in-line process analytical technology.^11^ To accelerate translation of this work to broader application in the pharmaceutical industry, benchmarking and standardization of the method is needed; hence an interlab comparative study on the benchtop NMR *R*_2_(^1^H_2_O) measurement was undertaken.

In this pilot study, an identical collection of samples containing model liquid dosage forms of pharmaceutical products was distributed to participating laboratories, and each lab performed low-field benchtop *w*NMR measurements on all samples using their own instruments. The primary goal was to assess the reliability, reproducibility, and information content of *R*_2_(^1^H_2_O) measurements for characterizing pharmaceutical products, such as biotherapeutic protein products and vaccines, across different laboratories with different operators, hardware, and software. A secondary goal was to identify and evaluate the sources of variability and their effects on measured *R*_2_(^1^H_2_O) values, which would serve as both feedback for method harmonization and guidance for future benchtop NMR interlaboratory studies.

## Experimental Section

### Sample Selection

The liquid pharmaceutical model samples selected for this study were: solutions of NIST reference monoclonal antibody (mAb) (NISTmAb, primary standard (PS) 8670 supplied by NIST) as a model for therapeutic protein products, and aluminum adjuvant suspensions of aluminum hydroxide (AH) gel, Alhydrogel^Ⓡ^ (Brenntag), and aluminum phosphate (AP) gel, Adju-Phos^Ⓡ^ (Brenntag), as models for aluminum-adjuvanted vaccines. We have previously demonstrated that in case of nonfunctionalized particles made of polymers with magnetic susceptibility close to water, *w*NMR is not sensitive to particle size or concentration.^12^ Since polyfluorinated polymers are known to have magnetic susceptibilities close to water,^13^ ethylene tetrafluoroethylene (ETFE) polymer particles were included in this study as negative controls. Indeed, our preliminary measurements of NIST ETFE reference material (ETFE, RM 8634 supplied by NIST) confirmed the independence of *R*_2_(^1^H_2_O) of the particle size or concentration. A single vial of an actual pharmaceutical product, Ferrlecit^Ⓡ^ (Sanofi), containing a sodium ferric gluconate complex with fast water relaxation, was supplied as a standard reference with its expected *T*_2_(^1^H_2_O) at 25 °C given in the study protocol.

### Sample Preparation

Samples were prepared by the IBBR study organizers, which participated in the study as one of 7 laboratories. A shipment of 24 samples was mailed on frozen cold packs to the other 6 participating laboratories, and a second shipment of Ferrlecit^Ⓡ^ was shipped at ambient temperature, according to the drug label storage requirements. Custom sample kits with foam inserts (Foam Factory) were packed inside boxes with thermal insulation panels (ULINE) designed to keep samples cool for up to 3 days to accommodate any potential delays due to international shipping destinations. Dry ice was not used because AP and AH samples would be damaged by freezing. Temperature excursion recorders (ULINE) were included to ensure the samples had not frozen.

Upon receipt of the sample shipment, one lab reported visual observations of frozen vials, and that lab and another lab both reported the temperature recorder indication of reaching freezing (below 0 °C) temperatures. Therefore, a new additional sample kit containing 19 vials was prepared and shipped to all participants including all samples, except for 5 NISTmAb samples that would have been undamaged with freezing and could be measured from the first kit (see Methods in Supporting Information). This new shipment excluded the thermal insulation panels and used a combination of refrigerated cold packs and frozen cold packs.

The 24 samples (0.5 mL volume each) were aliquoted into 2 mL (15 mm O.D.) glass amber vials (Thermo Fisher, ASTM E 438, USP Type I) with septa and crimp-sealed aluminum caps (Thermo Fisher). Individual vials were labeled with an alphanumeric code, concealing the content of each vial from the study participants, except for the IBBR team (see the Methods in Supporting Information). While sample identity was blinded to the participating laboratories, the different storage temperatures and handling requirements for each sample were provided. The recommended long-term storage temperature for NISTmAb is −80 °C, but it can tolerate several freeze/thaw cycles and refrigerated temperature storage for about a month.^14^ By contrast, AH and AP samples can be stored at room temperature or refrigerated but not frozen.^15,16^

The stock solutions of AH and AP were 10 and 5 mg/mL, respectively. These stock solutions were serially diluted 1:1 with ultrapure water (18.2 MΩ cm) to generate 6 AH sample concentrations of 0.31, 0.63, 1.25, 2.5, 5.0, and 10.0 mg/mL and 5 AP sample concentrations of 0.31, 0.63, 1.25, 2.5, and 5.0 mg/mL. In addition, one intentionally frozen vial of AH and one frozen vial of AP at the 1.25 mg/mL concentration were prepared in a 15 mL plastic tube by placing in a lab freezer at approximately −18 °C overnight (18 h). It was then thawed before aliquoting 0.5 mL into vials, such that one frozen AH vial and one frozen AP vial were included in each sample kit, which were to be compared with the same concentration (1.25 mg/mL) undamaged/unfrozen sample of AH and AP.

NISTmAb samples were prepared from 100 mg/mL stock solutions and were diluted with buffer (25 mM Histidine, pH 6.0) to achieve 5 sample concentrations of 1, 10, 25, 50, and 100 mg/mL and a sample of buffer alone. Likewise, ETFE samples were prepared from a stock of approximately 100,000 particles and were serially diluted 1:1 with diluent (0.02% sodium azide and 0.02% Triton X-100 solution in ultrapure water) to generate a set of 5 ETFE samples consisting of 6250, 12,500, 25,000, 50,000, and 100,000 particles. For all sample types, a larger volume at each concentration was prepared before aliquoting 0.5 mL into sample vials. An additional empty vial with septa and cap was provided for the purpose of testing cap removal procedure for those laboratories that transferred contents out of original vials into a standard 5 mm NMR tube.

### Study Protocol

Enclosed in the shipment of samples to all laboratories was the study protocol (see Methods in Supporting Information), which included instructions for sample handling and storage temperature, guidelines for *R*_2_(^1^H_2_O) data collection, and, if needed, directions for sample transfer to 5 mm glass NMR tubes for NMR instruments with bore sizes too small for intact vials.

Samples were to be inverted gently 20 times upon removal from storage (4 °C) and again gently inverted 20 times after equilibration to the instrument’s probe cavity temperature (minimum of 40 min) just prior to acquisition of the data. Inversion of the samples was important to ensure that the suspensions (AP, AH, ETFE) were fully dispersed at the start of the measurement. Since the sample contents were unknown to the participants, all the samples were inverted as a step in the measurement protocol. Guidelines for data collection in this pilot interlab study allowed for participants to adjust settings that best suited their instrument, within the following boundaries: measure the *R*_2_(^1^H_2_O) of each sample (either directly in the supplied sealed vial placed in the NMR instrument, or in a 5 mm NMR tube after sample transfer) at the same temperature using a Carr–Purcell–Meiboom–Gill (CPMG) pulse sequence and keeping the same interpulse delay time, τ, for measurements of all samples (e.g., τ = 500 μs). All laboratories in this study opted to measure at a single sample temperature between 25 °C and 30 °C.

### Data Collection

Each of the participant laboratories measured all the samples using their own benchtop NMR instrument (see Methods in Supporting Information) and for each participating Lab, the same instrument was used to measure the transverse relaxation time, *T*_2_, using the Carr–Purcell–Meiboom–Gill (CPMG) pulse sequence, for all the samples. The inverse of *T*_2_ is the relaxation rate *R*_2_: *R*_2_ = 1/*T*_2_.

The CPMG pulse sequence used consisted of a delay time (sometimes called relaxation delay (RD)), followed by a 90° pulse and then a string of *n* number of 180° pulses spaced by time, 2τ, and repeated for *m* number of scans (i.e., [RD–P90°–(τ–P180°−τ)_*n*_]_*m*_). Depending on the instrument or pulse sequence used, a time-domain- or frequency-domain-type CPMG experiment was conducted (see Methods in Supporting Information).

The following data collection settings were kept the same for all the samples in each Lab: the set (sample) temperature, the number of scans (also called transients or points), the interpulse delay (τ), the time (in μs) between the 90° pulse and 180° pulse in the CPMG pulse sequence. Laboratories adjusted their parameters to best suit their instrument, the details of which can be found in the Methods section of the Supporting Information. The sample temperatures used by the laboratories ranged from 25 to 33 °C, a slightly wider range than the protocol set forth (25 °C–30 °C). The number of scans used by the Laboratories ranged from 4 to 32, contributing to the overall experiment time per sample per lab ranging from 1 to 20 min. Laboratories used τ values ranging from 250 μs to 6000 μs.

### Data Analysis

A total of 267 CPMG measurements were submitted from the seven Laboratories. Representative data from each lab are shown in Figures S1 and S2. Each lab submitted its study data via email or a webform to the team at IBBR. Aside from the IBBR team, participants could not view data from other laboratories until data acquisition was completed by all participants in the study. The IBBR-UMB team compiled the submitted data from all sample measurements into a single multisheet Excel file per participant, with one measurement per sheet and an extra sheet with metadata containing parameters used. The IBBR-UMB team anonymized the submitted data by assigning a lab ID (e.g., Lab1, Lab2, etc.) to each participant so that the instrument maker was not revealed during the analysis of results by the IBBR-NIST team. The study data and associated processing by NMRPipe software and analysis scripts are available for download.^17^

A given relaxometry measurement was recorded in either two columns, specifying time (in seconds) and corresponding echo signal intensity (in arbitrary units), or was recorded as quadrature NMR measurements in three columns, specifying time (in seconds), real and imaginary components of the corresponding echo signal intensity, depending on the lab. Each spreadsheet of data was converted to a comma separated values (CSV) file, with one file for each spreadsheet (each sample measured) so that they could be input into the NMRPipe software for analysis by NMRPipe software tools.^18^

Raw data that was submitted from each participant was converted to complex time-domain format, with the imaginary part set to zero for measurements provided as real-only (two columns) data. Single exponential fitting was performed using the “modelXY” nonlinear least-squares model fitting application in NMRPipe, with the following model:

1 $$S_{obs}\left(t\right)=A\;\exp\left(-\alpha t\right)+b$$

where *S*_obs_(*t*) is the observed time-dependent echo signal intensity, *A* is the signal amplitude equal to the echo signal intensity at *t* = 0, α is the decay rate (s^–1^), which in this case specifically is the water proton transverse relaxation rate, *R*_2_(^1^H_2_O), *t* is time (s), and *b* is an offset correction to compensate for signals that decay to nonzero values.

Given the nature of the samples, it was expected that data from all laboratories predominately consisted of single-exponential signal decays, and so the residual of a single exponential fit should reveal whether a given sample is exhibiting more complex multiexponential relaxation behavior. However, in order to discern this, it was necessary to identify other sources of variation in the data such as random thermal noise and systematic measurement artifacts, in particular, phase distortions (Figure S3) and eddy current effects (Figure S4). In all cases, the impact of these artifacts was small, changing extracted relaxation values by less than 5%, as in the example shown in Figure S5. The details of these analyses and the compensating corrections applied are described in the Methods section of the Supporting Information.

The extracted *R*_2_(^1^H_2_O) values from artifact-corrected data for each sample type (NISTmAb, AH, AP, or ETFE) from each lab were plotted as a function of concentration and fit using OriginPro 2022 software. The slope of the linear dependence of *R*_2_(^1^H_2_O) on concentration is called relaxivity,^19^ which is a measure of efficiency of a solute in a given set of conditions to affect the relaxation of the (primarily) water protons in the solution,

$$R_{2}{(}^{1}H_{2}O)=R_{2,0}{(}^{1}H_{2}O)+r_{2}\times C$$ 2

where relaxivity, *r*_2_, is the slope (in (mg/mL)^−1^ s^–1^), *R*_2,0_(^1^H_2_O) is the *y*-intercept, and *R*_2_(^1^H_2_O) is the observed *R*_2_ of water protons at sample concentration, *C*.

The comparison of AH and AP samples that were intentionally frozen and those that were unfrozen was achieved by a calculation of the ratio of the *R*_2_(^1^H_2_O) of frozen Al­(III) adjuvant to *R*_2_(^1^H_2_O) of unfrozen Al­(III) adjuvant, or a percentage change as follows,

3 $$\%R_{2}{(}^{1}H_{2}O)\;change=\left[1-(R_{2}{(}^{1}H_{2}{O)}_{frozen}/R_{2}{(}^{1}H_{2}{O)}_{unfrozen})\right]\times 100\%$$

where the % change conveys the detection sensitivity of *R*_2_(^1^H_2_O) experiment at detecting the frozen outlier sample from the unfrozen sample (Tables ST6 and ST7).

The coefficient of variance (CV) for comparisons between the relaxivities of all seven laboratories was determined by the following equation,

4 CV=(σ/μ)×100%

where σ and μ are the standard deviation and mean, respectively, of the 7 sets of relaxivities from all 7 laboratories.

### Study Participants

There were seven total participants (“Labs”) in this study; therefore, there were seven sets of data. Six sample kits were shipped to participating laboratories, and the seventh was used by the IBBR Team. Aside from IBBR, participants were from five vendors: Bruker Biospin Corp., Magritek Inc., Nanalysis Corp., Oxford Instruments Magnetic Resonance, and Resonance Systems GmbH, with two different locations from Bruker Biospin Corp., participating as two separate laboratories. Additional information about the instruments that each participant used in this study can be found in the Methods section in Supporting Information.

## Results and Discussion

Many aspects of benchtop NMR, such as its compact size, simplicity, and information content, make it well suited to be adopted for routine QC/QA of drug substances and drug products. This interlaboratory study allowed for the testing of a range of approaches for measuring *R*_2_(^1^H_2_O) using benchtop NMR systems with different hardware and software configurations and designs, as well as a broader examination of the critical aspects of the application of the method that affect the data quality. Given the range of different benchtop NMR instruments used by the study participants, a less prescriptive approach, as compared to interlab studies involving more similar instrument configurations and methods, was used in this pilot study as a way to gather both quantitative data of benchtop NMR measurements for liquid pharmaceutical products, but also qualitative information about key variables in the application of the method to control. The results highlight the capabilities of *R*_2_(^1^H_2_O) in characterizing a variety of samples and also inform the design of reproducible protocols for practical use.

Sample kits (Figure 1) containing 25 vials of samples, whose contents were blind to participant laboratories, were shipped to 6 of the 7 laboratories, with an unblinded, unshipped sample kit used by the IBBR group. Different concentration samples included sets of NISTmAb, Alhydrogel^Ⓡ^ (AH), and Adju-Phos^Ⓡ^ (AP) to serve as model liquid dosage forms of two major types of biological products: therapeutic proteins, such as monoclonal antibodies (mAbs), and aluminum adjuvanted vaccines. Monoclonal antibody therapies have been hugely successful, with over 122 approvals in the US and around the world.^20^ Over 30 currently licensed vaccines in the US are aluminum adjuvanted, with antigen adsorbed to aluminum oxide particles.^21^ One set of NIST ethylene tetrafluoroethylene (ETFE) particle samples was included as controls and Ferrlecit^Ⓡ^, a marketed drug product, was included to serve as a 90° pulse length calibration standard.

**Figure 1:**
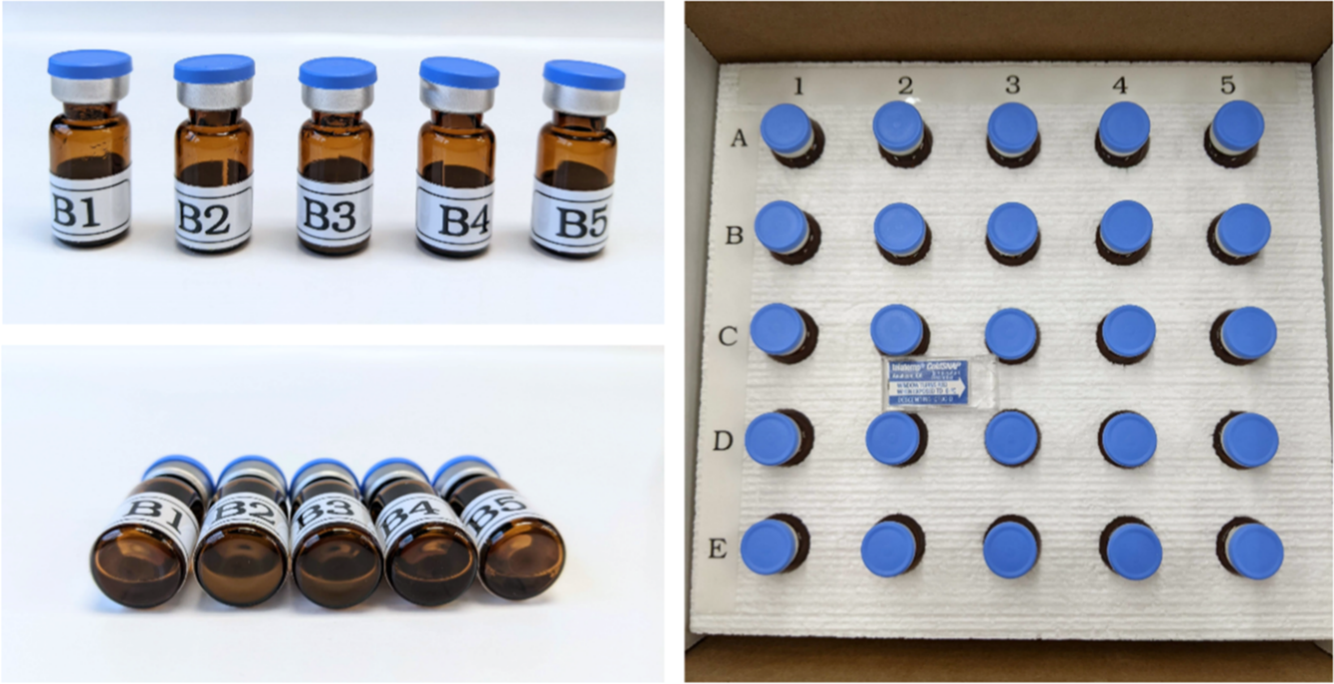
Samples were prepared in 2.0 mL amber glass vials and crimp sealed with septa and aluminum caps. Labels on the outside of the vial purposely obscured the view of the 0.5 mL liquid sample to imitate many liquid pharmaceutical vial labels. Sample contents were blinded.

### Assessment of Raw Data Variability

Among the seven Laboratories, the benchtop NMR instruments were operated in either time-domain or frequency-domain, at a ^1^H resonance frequency range of 17–80 MHz, with sample temperatures ranging from 25 to 33 °C, and a CPMG interpulse delay time (i.e., 90°–(τ–180°−τ)_*n*_ pulse separation), τ, ranging from 250 to 6000 μs. Additionally, five of the seven Laboratories measured the samples in the original sealed and capped glass vial (noninvasive measurements), while two Laboratories transferred the entire sample from the original sealed vial to a standard 5 mm NMR tube (invasive measurement). The measurement time of the experiment for each sample also varied from 1 to 20 min. These variables led to differences in the raw decay, such as the representative measurements shown in the left panel of Figure 2 for the same 25 mg/mL NISTmAb sample from each lab. Additional example measurements for a concentration range of NISTmAb samples are shown in Figure S1, with corresponding Fourier representations of these measurements shown in Figure S2, and measurement parameters are given in Table ST1. Efforts were made to identify potential sources of variability inherent in the raw data prior to model fitting in order not to propagate this variability in the extracted relaxation values.

**Figure 2:**
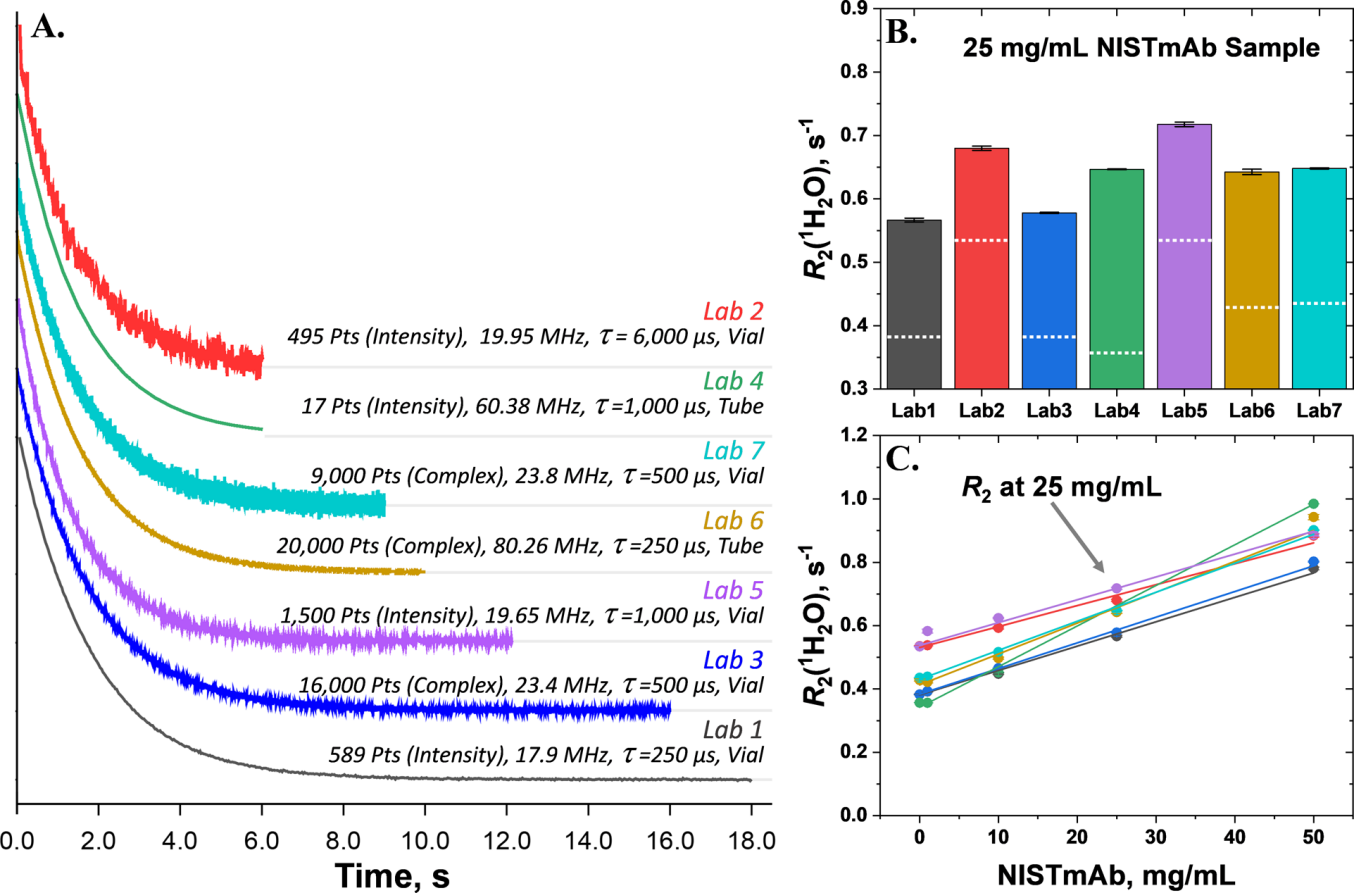
(A)­The offset-corrected relaxation curves of a representative sample (25 mg/mL NISTmAb) for each of the 7 participant laboratories in this study (curves vertically separated for clearer viewing). Measurements were performed according to each lab’s standard operating procedures for their instruments. (B) Bar graph showing the corresponding relaxation values extracted from single exponential fitting of the decay curves, along with relaxation values extracted in the same way from buffer samples (0 mg/mL mAb) indicated with a white dotted line. (C) The slope of the linear fit, or relaxivity, of the *R*_2_(^1^H_2_O) values for NISTmAb samples in this study, was determined for each lab (Table ST2). Note that error bars for exponential fits are narrower than the data points themselves.

An examination of the raw data identified and quantified three potential sources of error when measuring the model pharmaceutical samples by benchtop NMR. One potential source of systematic error is oscillations due to eddy currents within the instrument, especially those arising from the aluminum cap of vials in the noninvasive measurements. Such eddy currents contribute a much faster relaxing component (sub millisecond time scale) to the overall *R*_2_(^1^H_2_O) and depend to a greater or lesser degree on the instrument configuration or positioning of the sample in the instrument. Most pharmaceutical products in liquid dosage form contain rubber septa and aluminum caps. If noninvasive benchtop NMR measurements are an end goal, the degree that these eddy currents from the aluminum cap or instrument configuration contribute and/or interfere with the overall *R*_2_(^1^H_2_O) is relevant to understand, quantify, and potentially mitigate through protocol or configuration adjustments. The vials used in these experiments are comparable to those used in marketed pharmaceutical products, and an in-depth analysis of this potential source of error indeed showed a small contribution from eddy currents (Figure S4). To the affected raw data, fitting and subtracting an exponentially damped sinusoidal model of this eddy current contribution was able to compensate for the effect. The correction has only a small impact, as shown in the example of Figure S5, where the *R*_2_(^1^H_2_O) values changed by an RMS of only ∼3.6% when the correction was applied. It is worth noting that because the eddy current signal typically decays much faster than that of the water, it can be readily identified and corrected in *w*NMR measurements of liquid pharmaceutical samples, which are primarily aqueous.

The second identified potential source of error was attributed to phase deviations of the imaginary contribution of the echo decay. Small phase distortions may arise after many 180° pulses of the CPMG pulse sequence to a degree that depends on the field inhomogeneity and the transmitter/receiver coil of the instrument. While some laboratories submitted their data as a reconstructed echo signal intensity decay, other laboratories submitted complex data with real and imaginary components. Upon close inspection, the phase deviations of the imaginary component of the echo signal decays were found to be very small (Figure S3) and the effect of the phase correction on the extracted *R*_2_(^1^H_2_O) values compared to uncorrected was minimal, with an RMS of much less than 0.1% (Figure S6).

The third identified potential source of error is that of artifact signals from AC power sources, which take the form of constant oscillations in the relaxation measurement. Since these oscillations are typically small compared to the thermal noise, they are more or less invisible in the raw relaxation data and can only be revealed by Fourier transform, where they show up as spikes (Figure S2). Since these oscillations are small and centered at zero intensity, they are expected to affect exponential fitting results in a way comparable to thermal noise. Along these lines, AC power artifacts could confound thermal noise estimates made directly from the relaxation data, since the apparent thermal noise is actually a combination of thermal noise and AC oscillation artifacts.

These sources of error inherent in the raw data are minor contributors to the overall variation seen in the relaxation values from different laboratories. Estimates of these contributions of random and systematic noise for the different laboratories are shown in Figures S7–S10, and Figure S11 shows an example error analysis illustrating the effect of departure from single exponential behavior on estimates of *R*_2_(^1^H_2_O). In cases where data were affected by eddy currents and phase distortions, corrections for both were successfully applied, and the overall systematic distortion of intensity was small (generally less than 2%). The random noise was generally the largest contributor to variation in intensity. Therefore, the majority of the variation between the *R*_2_(^1^H_2_O) estimates from the single exponential fitting of the decays (e.g., top right of Figure 2) from different laboratories can be attributed to factors beyond these small systematic sources of measurement error.

### Variability Attributed to Operation

What we have termed operational variability broadly includes human error, operating procedures (e.g., calibrations, temperature, and instrument settings), selected CPMG parameters, and the type of CPMG pulse sequence (i.e., standard time-domain or frequency-domain CPMG) that can differ from one lab to another. Briefly, beyond operational variability, shipping cannot be excluded from the possible factors contributing to variability as these samples were shipped to seven different locations in four countries in North America and Europe, but evaluating or quantifying the effects of shipping was beyond the scope of this study.

As with previous interlaboratory NMR studies, there were occasional operator errors in sample and data handling.^22^ These included laboratories accidentally submitting data for one vial twice while missing another vial (2 out of 7 laboratories) and laboratories failing to invert samples before measurement (2 out of 7 laboratories). These problems were identified during the data analysis phase of the study, where data submission errors could be corrected, but it was not possible to remeasure samples that had not been shaken. In other cases, standard operating procedures needed to be optimized for the sample vial. For example, the data for one lab were much noisier than expected, and after discussions, it was discovered that the sample height should have been adjusted to accommodate the small 0.5 mL volume sample. To test that hypothesis, a new set of NISTmAb samples was sent to that lab for remeasurement with the optimized operational procedure (i.e., sample height). Adjusting for the sample height optimized the detection angle of the sample in the probe for that instrument, which significantly improved the signal-to-noise ratio, and therefore, the resulting relaxation rates better characterized each sample.

The results among all the 7 laboratories showed that for a given concentration of NISTmAb, the absolute *R*_2_(^1^H_2_O) values spanned a range, for example, a span of 0.23 s^–1^ for 1 mg/mL NISTmAb and 0.20 s^–1^ for 50 mg/mL NISTmAb (Figure 2, upper right panel), which might be expected from the differences between operating procedures and acquisition parameters. The lowest relaxivity for these samples was 6.61 × 10^–3^ (mg/mL)^−1^ s^–1^ and the highest was 12.82 × 10^–3^ (mg/mL)^−1^ s^–1^ for a span of 6.21 × 10^–3^ (mg/mL)^−1^ s^–1^ (Table ST2). The mean and standard deviation of these relaxivities is (8.77 × 10^–3^) ± (2.09 × 10^–3^) (mg/mL)^−1^ s^–1^ with a coefficient of variance (CV) of 23.8%, indicating that operational variables are a hindrance to reproducibility between laboratories, and there is room for improvement to achieve *R*_2_(^1^H_2_O) values in greater agreement across platforms.

The differences between laboratories, observed in the NISTmAb results, were likely due to individual laboratories’ parameters, such as the number of scans or transients, the number of echoes or points, and interpulse delay, τ. The mode of data collection, either in time-domain (TD) or frequency-domain (FD), using a CPMG pulse sequence to measure *R*_2_(^1^H_2_O), collects data as echo signal intensity decay or free-induction decay, respectively, and may introduce differences when comparing between different modes (Figures SM1 and SM2). Lab4 (green) data has a notably higher relaxivity than all other laboratories, with a striking difference of only 17 points acquired compared to the hundreds or thousands from other laboratories because of the different modes of CPMG experiment. It could be possible that the points for these measurements, which were hand selected, albeit with blind samples, did not capture the full extent of the relaxation decay (signal not fully returning to zero). However, data from this lab fits well to an exponential model as expected, indicating that the main reason for the difference in *R*_2_(^1^H_2_O) is inherent in the different modes of CPMG experiment used.

Also of note, while Lab4 (green) and Lab6 (gold) both use instruments with higher magnetic field strengths compared to the other laboratories and measured samples in 5 mm tubes instead of the provided sealed vials, measured *R*_2_(^1^H_2_O) values between these two laboratories differed, indicating that even with matched field strength and sample format, other factors appear to be influencing *R*_2_(^1^H_2_O) values. This corresponds with other work that shows only a shallow dependence of transverse relaxation on magnetic field strength.^23^ Here, the critical differences between these two laboratories were the number of points or echoes, the τ value, and mode of CPMG pulse sequence.

The NISTmAb measurements of the study were used to gauge the robustness of the *R*_2_(^1^H_2_O) as a measure of sample concentration while allowing for each lab’s normal operating procedures to be followed. The results from all 7 laboratories reliably reported positive linear slopes of *R*_2_(^1^H_2_O) with increasing concentration of NISTmAb. The results also show that the *R*_2_(^1^H_2_O), as a dynamic property of the water molecules in a particular sample environment, is not the same across different platforms with variable instrument properties and acquisition parameters. These differences could be addressed by using calibration standards, such as solutions of buffer and NISTmAb, to correlate measured values to standards for relaxivity.

### Measurement Comparison of Aluminum Adjuvant and ETFE Relaxivities

Increasing in measurement complexity, aluminum adjuvants are suspensions that settle over time and need to be fully suspended before and during vaccine vial filling, resuspended before patient injection, and in this case fully suspended before benchtop NMR measurement. Although this study focused solely on fully suspended aluminum adjuvants, the critical measurement of the extent of aluminum adjuvant resuspension can be quantified using *w*NMR.^24^ These aluminum oxide particles also have a high magnetic susceptibility contrast with water molecules, which results in a greater relaxation efficiency, resulting in steeper slopes and larger *R*_2_(^1^H_2_O) values for higher concentrations.

The concentration ranges in terms of Al­(III) of the two sets of adjuvant samples in this study are different, stemming from different commercially available stock concentrations of each, AH being higher than AP. Results for these adjuvant solutions reveal that sample handling procedures are crucial for systems that need to be fully suspended. The linear fits of the *R*_2_(^1^H_2_O) of six AH samples (0.3–10.0 mg/mL of Al­(III)) indicated that out of the seven laboratories, two laboratories appeared to be outliers (Figure 3A). The *R*_2_(^1^H_2_O) slopes from five laboratories are relatively close to each other, ranging from 0.71 to 1.08 for a span of 0.37 (mg/mL)^−1^ s^–1^ (Table ST2). The mean and standard deviation of the *R*_2_(^1^H_2_O) slopes, excepting the two outliers, is 0.93 ± 0.15 (mg/mL)^−1^ s^–1^ with a CV of 16.3%. Likewise, with the exception of two laboratories, the *R*_2_(^1^H_2_O) slopes for the AP samples (0.3–5.0 mg/mL of Al­(III)) are relatively close to each other (Figure 3B), ranging from 1.82 to 2.17 for a span of 0.35 (mg/mL)^−1^ s^–1^ (Table ST3). The mean and standard deviation of the *R*_2_(^1^H_2_O) slopes, except the two outliers, is 1.98 ± 0.15 (mg/mL)^−1^ s^–1^ with a CV of 7.7%.

**Figure 3:**
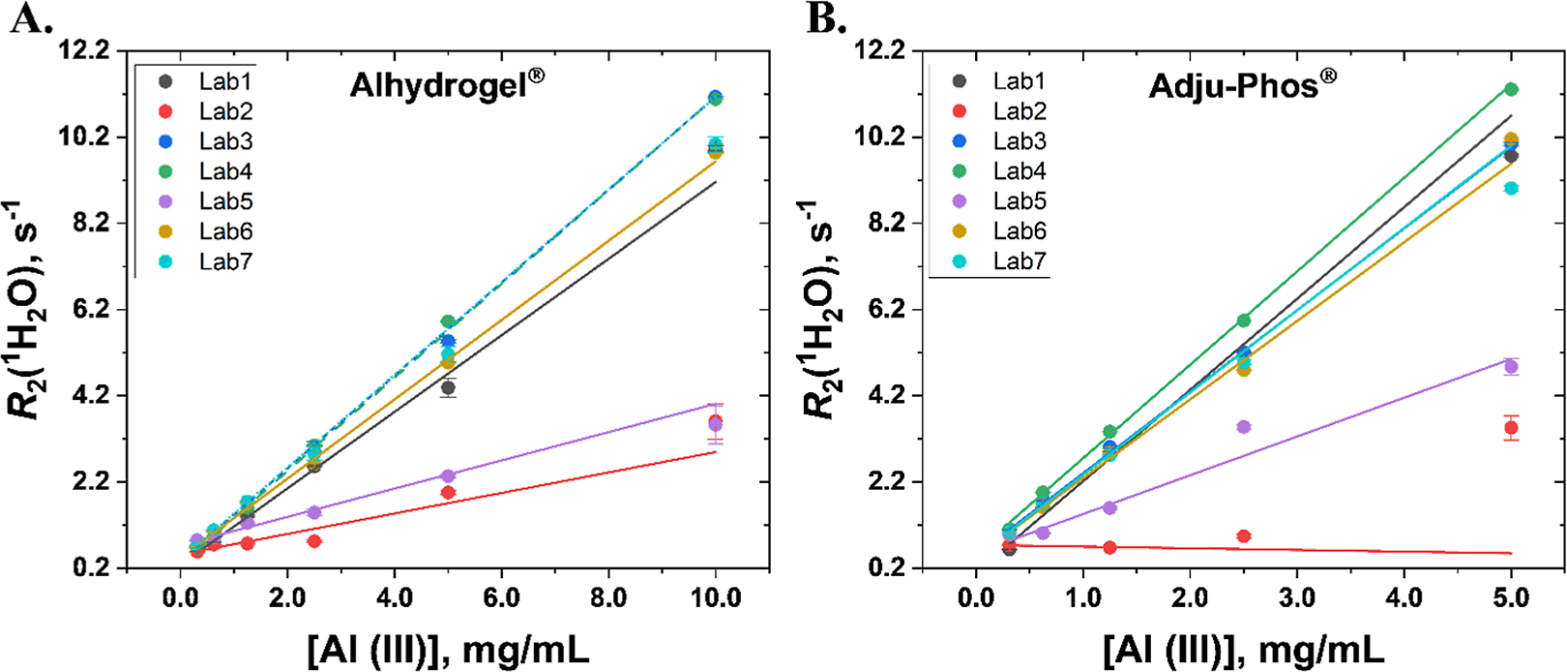
*R*_2_(^1^H_2_O) of (A) Alhydrogel^Ⓡ^ concentration series (0.3–10.0 mg/mL of Al­(III)) and (B) Adju-Phos^Ⓡ^ concentration series samples (0.3–5.0 mg/mL of Al­(III)) with linear fits of the concentration series per lab are shown for all seven laboratories, from which the relaxivities are derived from the slopes (Tables ST4 and ST5). Error bars are mostly within the size of each symbol and represent error estimates. For AH, Lab3, Lab4, and Lab7 appear as dashed lines with their linear fits of 1.076 mg/mL^–1^s^–1^, 1.085 mg/mL^–1^s^–1^, and 1.074 mg/mL^–1^s^–1^, respectively, overlapping. For AP, Lab3 and Lab7 linear fits, 1.895 mg/mL^–1^s^–1^ and 1.907 mg/mL^–1^s^–1^, respectively, also overlap.

These samples were blind to all the participant laboratories and later discussions revealed that the two outlier laboratories simply missed the instructions to invert the samples before measurement, explaining the outlier behavior. This also highlights the sensitivity of benchtop NMR toward the aluminum particle sedimentation, in part due to the magnetic susceptibility contrast between aluminum oxide and water noted above.^25^ Because of this effect, the settling of aluminum particles over the course of a longer CPMG experiment time may also contribute to differences between laboratory data. Comparing this operational variable, Lab2 (red) and Lab4 (green) reported longer experiment times of approximately 20 min (Supporting Information Methods), but Lab4 relative conformity with the other laboratories suggests measurement times in this range do not contribute substantially to changes in measured *R*_2_(^1^H_2_O) values.

NIST ETFE Particle suspension reference material, provided in different amounts but all in the same dispersion medium (0.02% sodium azide and 0.02% triton x-100 surfactant in water), served as a negative control since the *R*_2_(^1^H_2_O) measurement should not be sensitive to the ETFE particle material. Indeed, the change in number of ETFE particles had an *R*_2_(^1^H_2_O) slope of zero for all partnering laboratories (Figure S13 and Table ST3).

### Comparison of Freeze/Thaw Detection of Aluminum Adjuvants

Vaccines containing aluminum adjuvants should be discarded if they are damaged by experiencing freeze/thaw during shipment and distribution.^26^ Benchtop NMR is capable of detecting prior freeze/thaw events on thawed vaccines.^10^ In particular, it was observed that for both free adjuvants and marketed vaccines, prior freeze/thaw events cause a decrease in *R*_2_(^1^H_2_O).^10^ Freezing of aluminum adjuvants causes particle agglomeration that is irreversible. The larger aluminum particle size results in lower surface area overall and decreased water relaxation efficiency and thus a lower *R*_2_(^1^H_2_O).

Two AH samples (1.25 mg/mL of Al­(III)), one unstressed and one freeze/thaw stressed, and two AP samples (1.25 mg/mL of Al­(III)), one unstressed and one freeze/thaw stressed, were among the set of samples measured by each lab. The *R*_2_(^1^H_2_O) values of the freeze/thaw sample were compared to the *R*_2_(^1^H_2_O) of the unstressed sample plotted from each lab, with identical values indicating no change (Figure 4). It is worth noting that each lab detected a 10% or greater change, in *R*_2_(^1^H_2_O) upon an incidence of freeze/thaw for both AH and AP samples (Figure 4). It has been demonstrated that the *R*_2_(^1^H_2_O) values have a linear response to concentration^27^ (Figure 3), so, for practical purposes, the standard deviation of *R*_2_(^1^H_2_O) (measured) – *R*_2_(^1^H_2_O) (linear fit) as the estimate for the error in *R*_2_(^1^H_2_O), as indicated by dashed lines in Figure 4. The error thus encapsulates error from the instrument, handling, and/or operations (Tables ST4 and ST5), and confidence that measured values outside the range represent true sample outliers.

**Figure 4:**
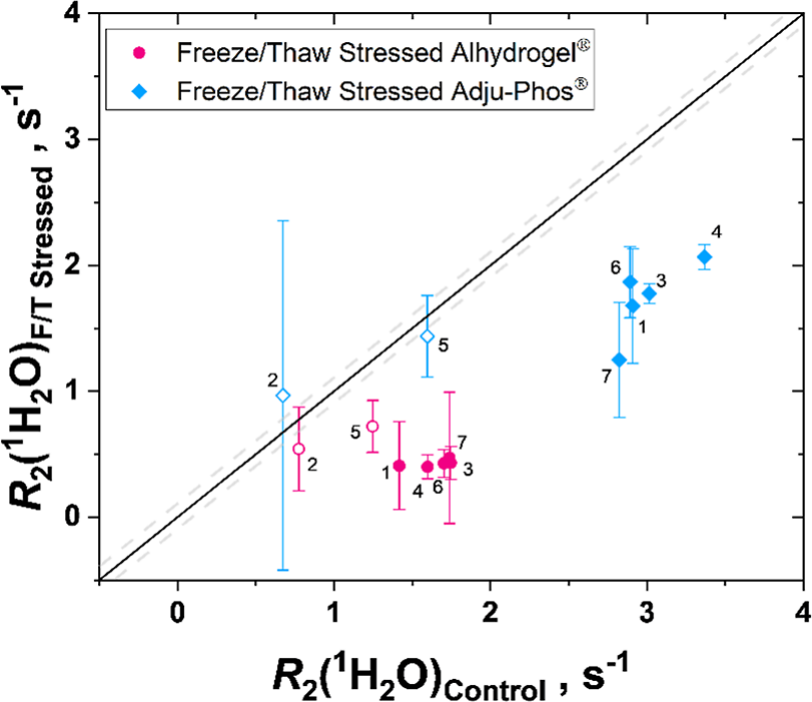
Ratio of *R*_2_(^1^H_2_O) of the F/T stressed adjuvant [1.25 mg/mL of Al­(III)] sample to the *R*_2_(^1^H_2_O) of the unfrozen adjuvant [1.25 mg/mL of Al­(III)] sample, for (A) Alhydrogel^Ⓡ^ and (B) Adju-Phos^Ⓡ^, reported from each partnering lab indicates that each lab detected a 10% (dashed line) or greater change in *R*_2_(^1^H_2_O) after an incidence of freeze/thaw. The standard deviation for each lab was derived from RMSD calculations from the measured *R*_2_(^1^H_2_O) compared to *R*_2_(^1^H_2_O) of the linear fit for each lab’s Alhydrogel^Ⓡ^ (Figure 3A) and Adju-Phos^Ⓡ^ data (Figure 3B). It is expected that the outlier data, indicated with hollow data points, is due to measurements that skipped the prescribed step of resuspension of the samples.

Although most laboratories observed a significant decrease of *R*_2_(^1^H_2_O) upon a freeze/thaw cycle, one lab observed an increase, instead of a decrease, of *R*_2_(^1^H_2_O) upon a freeze/thaw cycle of AP. The reason for this is not entirely clear but may possibly be due to incomplete or lack of resuspension of one or both of the measured adjuvant sample from the pair of samples. Regardless, the *R*_2_(^1^H_2_O) measurements of both AH and AP aluminum adjuvants by every participating lab were capable of distinguishing a standard quality aluminum adjuvant suspension vial from an aluminum adjuvant altered by a single freeze/thaw incident, despite 6 of 7 laboratories not even being aware that there would be a “damaged” sample for detection.

Many aspects of benchtop NMR, such as its compact size, simplicity, and information content, make it well suited to be adopted for the routine QC/QA of drug substances and drug products during development and manufacturing. This interlaboratory study allowed testing of a range of benchtop NMR instruments with different hardware configurations and software. It allowed for a broad examination of critical variables that affect the *w*NMR data quality, a key aspect of adapting benchtop *w*NMR measurements for use in pharmaceutical manufacturing. A less prescriptive approach was used in this study compared with studies that use the exact same hardware and protocols. Thus, this pilot study gathered both quantitative data of benchtop NMR measurements for liquid pharmaceutical products and qualitative information about key variables that could be controlled in a larger follow-on study. The results in this study highlight the capabilities of *R*_2_(^1^H_2_O) in characterizing a variety of samples and also informing the design of reproducible protocols for practical use.

## Conclusions

The goal of this study was to identify sources of variability in *R*_2_(^1^H_2_O) measurements, as a first step in the longer-term goal of establishing protocols and references for reliable and robust application of *w*NMR in the pharmaceutical industry. A critical finding of the study is that relaxation values for a given sample class and measurement configuration are highly linear with respect to concentration of an undamaged sample and that this holds true for all sample classes, instrument types, and relaxation measurement protocols included in the study. This suggests that a properly calibrated *w*NMR measurement can quantitatively confirm concentration as well as identify damaged samples by their outlier relaxation values. That said, the study outcomes have identified several sources of variability that inform the next steps in harmonizing benchtop NMR *R*_2_(^1^H_2_O) measurements such as parameter selection, calibration steps, and sample handling. These could be included in follow-on interlaboratory studies, to establish robust applications of the technique that will enable broad use as a tool for quality assessment in pharmaceutical development.

## Supplementary Material



## Data Availability

All raw data (as.csv files) and scripts are supplied at https://www.ibbr.umd.edu/groups/benchtop-nmr/data.

## ○

Eli Lilly and Co., 307 East Merrill, Indianapolis, Indiana 462221, United States
