# Supplementary material for: Multiplatform Benchtop NMR Interlaboratory Study of Model Liquid Dosage Forms of Pharmaceutical Products
Source: Anal Chem. 2026 Jan 5;98(2):1451–9. doi: 10.1021/acs.analchem.5c05487 (PMC12824986; doi:10.1021/acs.analchem.5c05487)
Supplement: Supplementary file 1 [file ac5c05487_si_001.pdf]

Multiplatform Benchtop NMR Interlaboratory Study of Model Liquid Dosage Forms of Pharmaceutical Products

Katharine T. Briggs[1†]*, Frank Delaglio[2†], Marc B. Taraban[1], Robert G. Brinson[2], Luke Arbogast[2,9], Brendan Lichtenthal[3], Matteo Pennestri[4], Robert Espina[5], Hector Robert[5], Juan F. Araneda[6], Paul Hui[6], Susanne D. Riegel[6], Kevin Nott[7], Leonid Grunin[8], Innokenty Nikolaev[8], Thomas Reininger[8], Y. Bruce Yu[1], John P. Marino[2]*

[1] Institute for Bioscience and Biotechnology Research, University of Maryland, Baltimore, Rockville, MD 20850 USA
[2] Institute for Bioscience and Biotechnology Research, National Institute of Standards and Technology, Rockville, MD 20850 USA
[3] Bruker BioSpin Corp. 15 Fortune Drive, Billerica, MA 01821 USA
[4] Bruker BioSpin Corp. Banner Lane, Coventry, CV4 9GH, United Kingdom
[5] Magritek, Inc. 103 Great Valley Pkwy, Malvern, PA 19355
[6] Nanalysis Corp. 1-4600 5 St. NE, Calgary, AB, Canada T2E 7C3
[7] Oxford Instruments Magnetic Resonance. Tubney Woods, Abingdon, OX13 5QX, UK
[8] Resonance Systems, GmbH Seestrasse 28, D-73230, Kirchheim/Teck, Germany
[9] Current Address: Eli Lilly and Co. 307 E. Merrill, Indianapolis, IN 462221 USA
†Katharine T. Briggs and Frank Delaglio contributed equally to this work.
*Corresponding Authors: kbriggs@rx.umaryland.edu, john.marino@nist.gov

Interlaboratory Study Protocol and Pre-Study Measurement

Description	Page
Interlaboratory Study Protocol...………...	S4
Preliminary data on NIST ETFE particles (pre- interlaboratory study)…..	S10

Supporting Information

**Methods**	**Description**	**Page**
	Samples measured in this Study……………………………………...	S11
	Sample kits shipped to each participant………………………………	S12
	Benchtop NMR Instruments Used in this Study……………………...	S13
	Time-Domain vs Frequency-Domain CPMG Experiment…….……..	S14
	Parameters Selected for CPMG Experiments by Each Lab…….........	S16
	Relaxation Data and Analysis…………………………....................	S17
**Methods Figures**		**Page**
Figure SM1	Time-domain measurement of transverse relaxation...........................	S14
Figure SM2	Frequency-domain measurements of transverse relaxation…………...	S15
**Equations**		
Eq. S1	Single exponential…………………………………………………….	S18
Eq. S2	Total signal contribution (**S_{total}**)……………………………………….	S19
**Supporting Figures for Main Text**		
Figure S1	Representative data from each lab…………………………………….	S21
Figure S2	Fourier transform of representative data from each lab..……………...	S22
Figure S3	Example phase correction of phase imperfection…...…………………	S23
Figure S4	Example data demonstrating eddy current distortion and correction....	S24
Figure S5	Representative $R_2(^1H_2O)$ data are plotted before and after sinusoid correction was applied………………………………………………...	S25
Figure S6	Representative data show the effect of phase correction on the extracted $R_2(^1H_2O)$ values…………………………………………………….	S26
Figure S7	Estimated signal contributions from Lab1, Lab3, and Lab6…………..	S27
Figure S8	Estimated signal contributions from Lab2, Lab5, and Lab7…………..	S28

Figure S9 Estimated signal contributions from Lab4…………………………......... S29
Figure S10 Example alpha decay rate parameter with random point deletion..……… S30
Figure S11 Example alpha decay rate parameter with systematic point deletion……. S30
Figure S12 Relaxivity of NISTmAb before and after optimizing detection angle…… S31
Figure S13 $R_2(^1H_2O)$ of NIST ETFE particles……………………………………….. S32

Tables

Table ST1 Measurement parameters by Lab………………………………………… S17
Table ST2 Relaxivities of NISTmAb ……………………………........................ S33
Table ST3 Relaxivities of NIST ETFE particles..…………………………………… S33
Table ST4 Relaxivities of Alhydrogel®……………………………………………... S33
Table ST5 Relaxivities of Adju-Phos®………………………………………………. S33
Table ST6 Detection of freeze/thaw damaged Alhydrogel® sample…..…………….. S34
Table ST7 Detection of freeze/thaw damaged Adju-Phos® sample…….…………… S34
Table ST8 Reported relaxation rates of Ferrlecit® …………………………...... S35
Table ST9 Table of Variables Identified in this Study………………………………. S36

References

S37

Methods: Interlaboratory Study Protocol

The details of the following protocol were discussed by all participants at an initial meeting and finalized by the IBBR study organizers. A hard copy of the study protocol was provided to each participant along with the shipment of samples. The contents of the study protocol are provided below (pgs. 4-9).

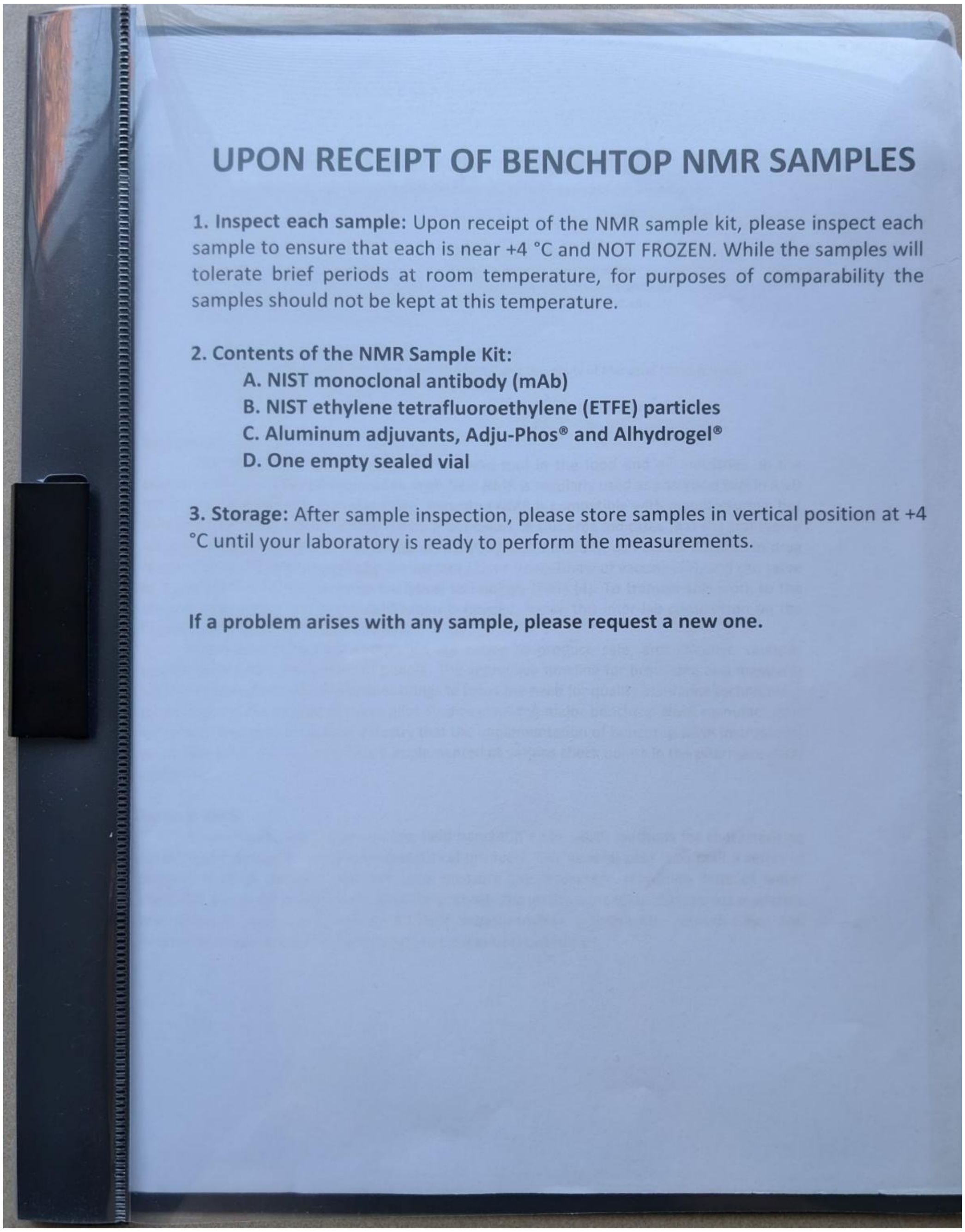

UPON RECEIPT OF BENCHTOP NMR SAMPLES

1. Inspect each sample: Upon receipt of the NMR sample kit, please inspect each sample to ensure that each is near +4 °C and NOT FROZEN. While the samples will tolerate brief periods at room temperature, for purposes of comparability the samples should not be kept at this temperature.

2. Contents of the NMR Sample Kit:

- **A. NIST monoclonal antibody (mAb)**
- **B. NIST ethylene tetrafluoroethylene (ETFE) particles**
- **C. Aluminum adjuvants, Adju-Phos® and Alhydrogel®**
- **D. One empty sealed vial**

3. Storage: After sample inspection, please store samples in vertical position at +4 °C until your laboratory is ready to perform the measurements.

If a problem arises with any sample, please request a new one.

Study Protocol

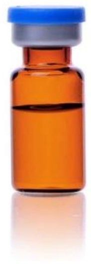

Each partner will be mailed the same 25-30 samples sealed in 15 mm O.D. glass vials along with instructions for sample storage, for conducting the measurement, and for reporting the data to NIST/UMB.

Sample Contents

Liquid dosage forms of pharmaceutical products include solutions, emulsions, or suspensions. The samples in this study will model liquid dosage forms of pharmaceutical products. The contents of the samples will be some combination of the following: NIST monoclonal antibody (NISTmAb), NIST ethylene tetrafluoroethylene (ETFE) particle suspension, aluminum hydroxide gel suspension, aluminum phosphate gel suspension. The samples will be labeled a number or letter so that the sample contents will be blinded. As with most liquid dosage forms, the main component of all the samples will be water. Each of the 25-30 sample vials will be approximately 0.5 mL in volume. A vial of Ferrlecit®, an iron carbohydrate drug product, will be supplied as a standard, along with the expected $T_2(^1H_2O)$ of the drug product at 25 °C. All samples are non-hazardous, and the sealed sample vials will be mailed to all partners.

Sample Storage

Upon receipt of the shipment of samples, each sealed vial in the carton should be visually inspected for evidence of vial freezing (*i.e.*, ice formed inside the vial), and then the samples should be swiftly placed into a refrigerator (2 °C to 8 °C). All samples should be stored at ≈ 4 °C whenever they are not being measured.

Upon receipt of the single vial of Ferrlecit®, which will be shipped separately, visually inspect the vial for signs of physical damage to the vial or contents (*e.g.*, cap dented, septum pierced, ice formed). Ferrlecit® should always be stored at (20 to 25) °C, or room temperature.

Sample Handling

At least 40 min before measuring samples, equilibrate the sample vial at the measurement temperature. Handle each vial by the aluminum cap to prevent fingers from warming the sample. Gently invert the sample 20 times (back and forth), trying not to produce bubbles and/or foam. Set the sealed vials on a benchtop surface, away from sunlight, until the samples to be measured equilibrate to the room temperature.

If the temperature of the room is different from the measurement temperature of the NMR, consider equilibrating the samples individually in the NMR for 40 min prior to a measurement, or placing samples in a water bath calibrated to the set temperature of the NMR instrument.

After the samples are temperature equilibrated and experimental parameters are set, retrieve the sealed sample vial (from the benchtop NMR bore, water bath or benchtop), grasp at the aluminum cap, and invert 20 times gently, again trying not to produce bubble and/or foam, and then place the sample into the instrument for acquisition of the measurement. This will ensure that suspensions are fully suspended for the $T_2(^1H_2O)$ measurement of the sample.

Data Collection

Harnessing the high concentration of the water component, each partner will measure the transverse relaxation time of the water protons, $T_2(^1H_2O)$, of each of the samples using a CPMG pulse sequence. The sealed sample vials are to be measured directly in the benchtop NMR instrument. The samples should be run at a single temperature between (25 to 30) °C, with 28 °C being optimal for this study. The 25-30 sample vials for analysis should be measured at the exact same temperature using the same primary instrument and should have the same inter-pulse delay (*e.g.*, 500 µs). Additional benchtop NMR instruments may also be used to measure all or a subset of the samples. If these additional measurements are done, the additional data sets should be reported with parameters and experimental details specific to the benchtop NMR instrument that was used.

Following the measurement of the sealed vials, each partner will transfer samples to a standard 4 mm or 5 mm NMR tube (see *Sample Transfer*) and again measure the $T_2(^1H_2O)$ of each sample.

Sample Transfer

If your benchtop NMR instrument can measure the materials in the provided sealed, unopened vials, complete the measurements with the sealed, unopened vials first, and then transfer samples from the vial to a standard 4 or 5 mm NMR tube. If possible, collect data using the 4 or 5 mm NMR tube on the same benchtop NMR instrument as was used for the sealed vials (see *Data Collection*).

In the absence of a 13 mm (cap size) hand decrimper decapper, the following is a protocol for transferring liquid sample from the sealed vial into an NMR tube. If an alternative method is used, the most important aspect of transferring samples is to gently invert the sample just before pipetting (minimizing bubbles) to ensure that suspensions are fully suspended. Keep all sealed sample vials at 4 °C until each vial is ready for cap removal and sample transfer to minimize time at room temperature.

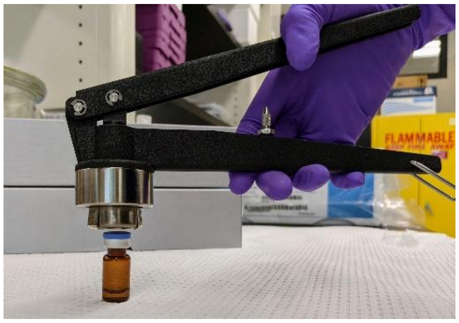

<table>
<tr>
<td><u>Materials:</u>
▪ Gloves
▪ Pipette and tips
▪ Razor blade or box cutter
▪ Needle-nose pliers
▪ Vial rack
▪ Sealed sample vials
▪ NMR tubes (5 mm or 4 mm) with caps</td>
<td>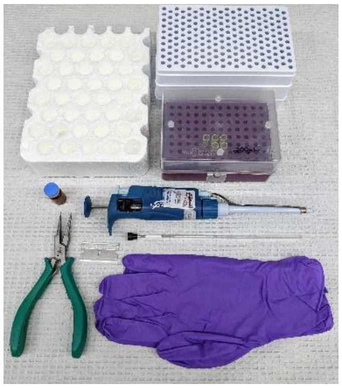</td>
</tr>
</table>

<u>Transfer Procedure:</u><br>1) Clean and clear a counter space. Place vials in rack.<br>2) Prepare clean and dry NMR tubes and caps for transfer.<br>3) Wear gloves.<br>4) Pop off colored cap.	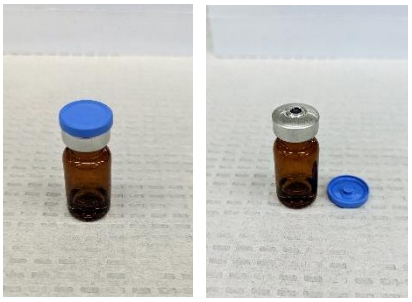
5) Use a razor blade (taking lots of care not to nick yourself) to press the blade into the aluminum rim to create a perforation line (without pressing too much into the grey rubber septum).	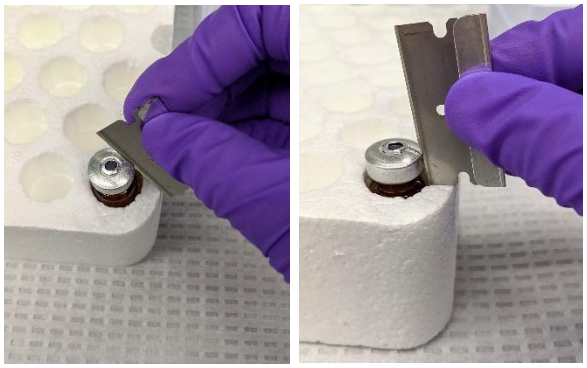
6) Once a perforation has been started, use the short edge of the razor blade (or a less sharp utensil) to pull off the inner ring at the top of the aluminum seal. Continue to pry the outer aluminum rim from the top.	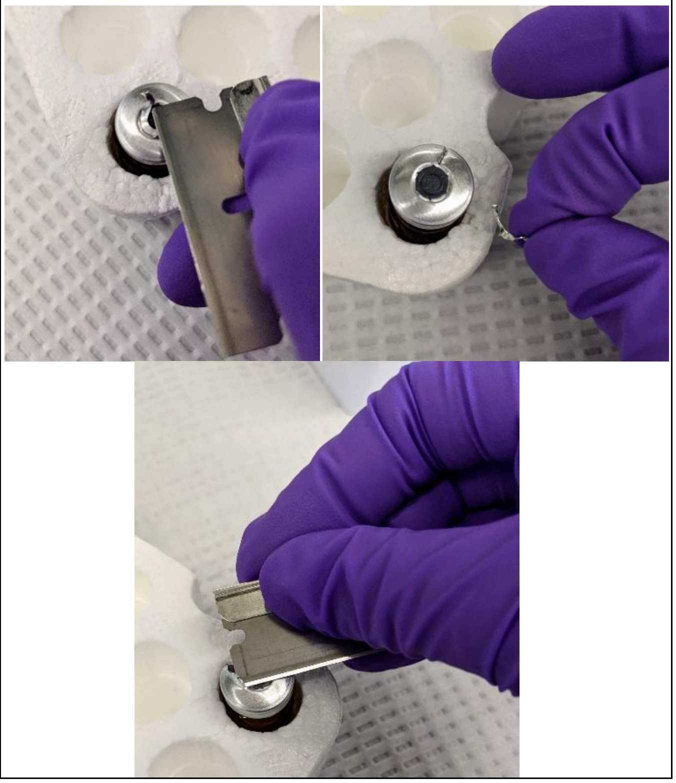

7) Once an edge has been pried up enough, use needle-nose pliers to pull off the seal without cutting fingers. Non-dominant hand can hold vial in place to prevent from tipping over.	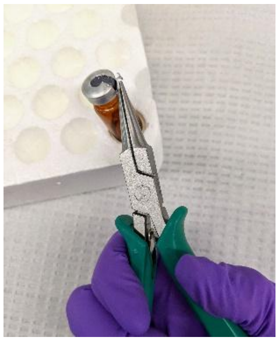 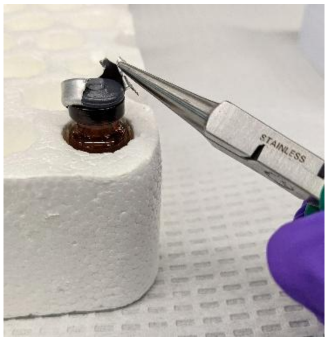
8) Keep septum seal in place while aluminum seal is completely pulled off. Wipe off aluminum remnants from septum.	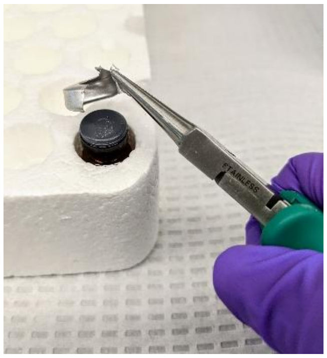 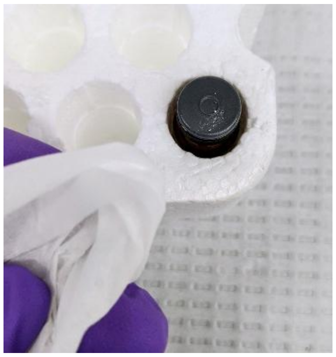
9) Hold the vial in hand, firmly pressing the septum at the top of the vial, and invert vial 20 times to resuspend suspensions.<br>10) Tap or lightly flick the vial so any liquid from underneath the cap slides down to the bottom of the vial.	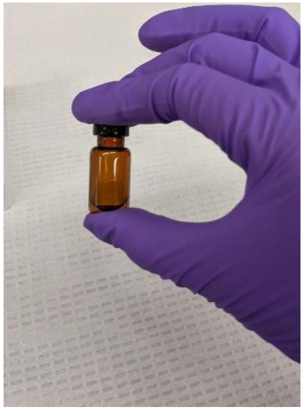 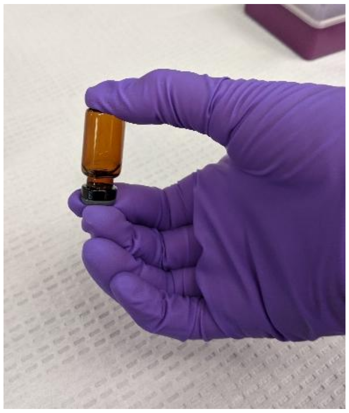
11) Set vial in rack. Remove septum. Pipette 0.5 mL liquid sample from vial to NMR tube. Check cap for remaining liquid and transfer this liquid too. Cap NMR tube.<br>12) Store NMR tubes at 4 °C.	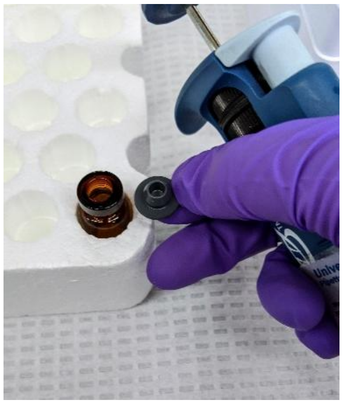 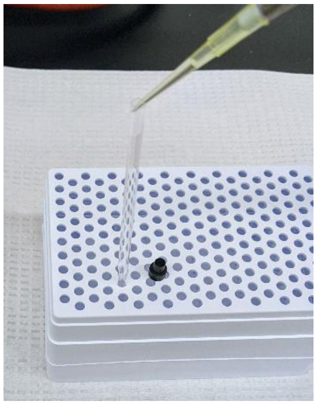

Samples in 4 or 5 mm NMR tubes should be brought to the measurement temperature and resuspended prior to data collection in the same way as the sealed vials (see *Sample Handling, Data Collection*).

Data Analysis

Once the data has been collected, partners will send the following data and information to the IBBR Team:

1) Instrument(s) specifications (*e.g.*, magnetic field strength, bore size, temperature inside the cavity).

2) Acquisition parameters.

3) Simple text format of the data (time, real, imaginary) for extracting the $T_2(^1H_2O)$ (*e.g.*, in an excel spreadsheet).

4) Extra information about how the experiment was conducted with respect to the pulse sequence, instrument parameters, sample preparation, and sample transfer.

The simple text format of the data will be anonymized by assigning each data set a generic name (*e.g.*, 'Lab 1', 'Lab 2', etc.). The published data will be presented without associating the data with the partners. The anonymized data will be made available to all partners.

Methods: NIST Ethylene Tetrafluoroethylene (ETFE) Preliminary Data

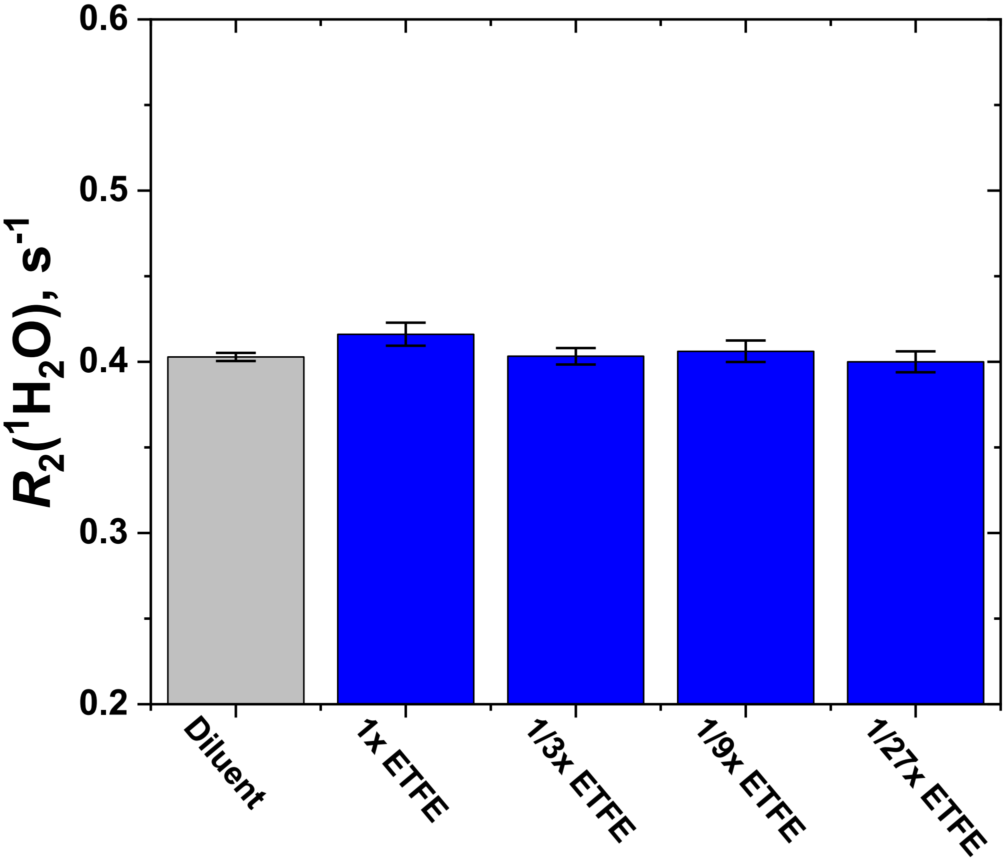

Preliminary data on ETFE particles during early discussions of study design, and prior to sample kit preparation, showed that the particles had little to no effect on the $R_2(^1H_2O)$. The diluent at left (grey) is compared to a dilution series of ETFE particles (blue), with only a very slight difference between the highest number of particles (1x concentration) and the diluent.

Methods: Samples Measured in this Interlaboratory Study

The contents of the 25 samples are revealed below, sorted by sorted by sample type.

Code	Type	Concentration	Units
A5	NISTmAb	0	(mg/mL)
B4	NISTmAb	1	(mg/mL)
D2	NISTmAb	10	(mg/mL)
E3	NISTmAb	25	(mg/mL)
D4	NISTmAb	50	(mg/mL)
C3	NISTmAb	100	(mg/mL)
C1	ETFE	6 250	(# particles)
A2	ETFE	12 500	(# particles)
D1	ETFE	25 000	(# particles)
C2	ETFE	50 000	(# particles)
B5	ETFE	100 000	(# particles)
A1	AP	0.31	(mg/mL)
E2	AP	0.63	(mg/mL)
B3	AP	1.25	(mg/mL)
D3	AP	2.5	(mg/mL)
B2	AP	5	(mg/mL)
D5	AP-Frozen	1.25	(mg/mL)
E4	AH	0.31	(mg/mL)
A4	AH	0.63	(mg/mL)
A3	AH	1.25	(mg/mL)
C5	AH	2.5	(mg/mL)
E1	AH	5	(mg/mL)
C4	AH	10	(mg/mL)
B1	AH-Frozen	1.25	(mg/mL)
Ferr	Ferr	12.5	(mg/mL)

Abbreviations: NISTmAb, NIST monoclonal antibody; AP, Adju-Phos®; AH, Alhydrogel®; ETFE, NIST ethylene tetrafluoroethylene particles; Ferr, Ferrlecit®.

Methods: Sample Kits Shipped to Each Lab

A. *Shipment #1* **B.** *Shipment #2*

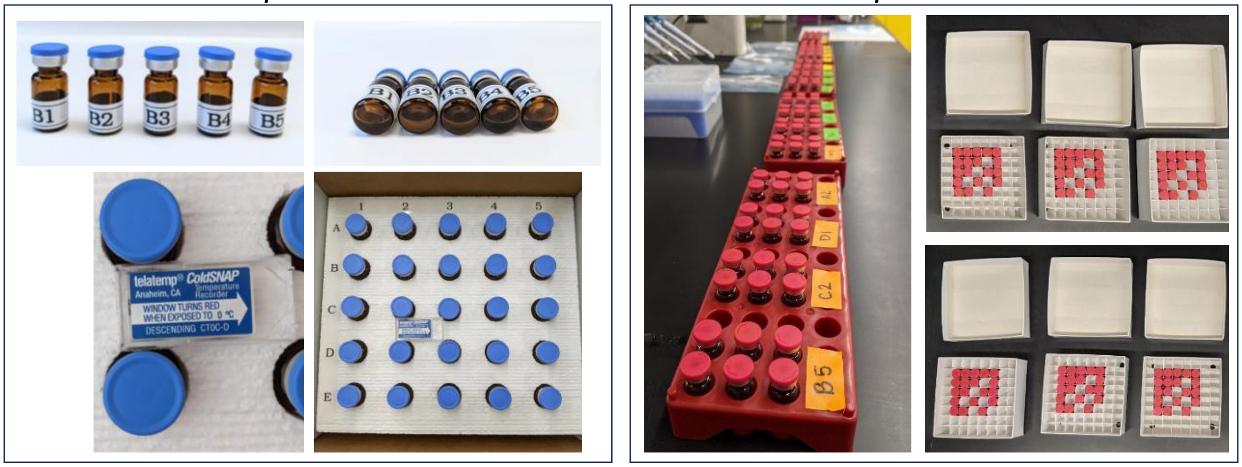

Sample kits had 25 amber capped vials containing 24 samples for the study and one extra vial for testing de-capping procedures. Two labs reported one or more samples were frozen upon arrival of Shipment #1 (**A**) and the freeze indicator having turned red. Shipment #2 (**B**) was packaged with fewer frozen cold packs and less insulation to prevent freezing; it contained 19 samples that replaced all the freeze-damage susceptible samples from the shipment #1. All labs used the 19 replacement samples from shipment #2 and the 5 NISTmAb samples from shipment #1 for this inter-lab study.

Methods: Instruments Used in this Study

Benchtop NMR instruments were used in the study. One participant offered two sets of data (two Labs) for a total number of data sets coming from 7 Labs.

Participant	Instrument
Bruker	Mq20 MiniSpec NF4885
Bruker	Mq20MiniSpec NF4028
IBBR	MQC+
Magritek	Spinsolve 80 Carbon
Nanalysis	60e
Oxford Instruments	MQC+
Resonance Systems	SpinTrack SB45

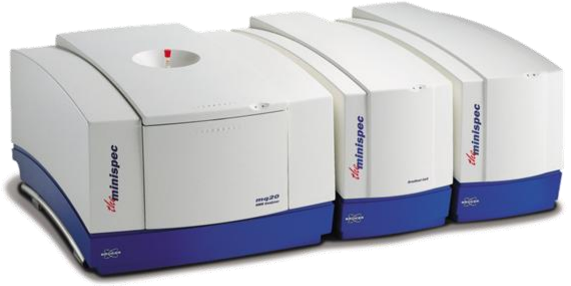

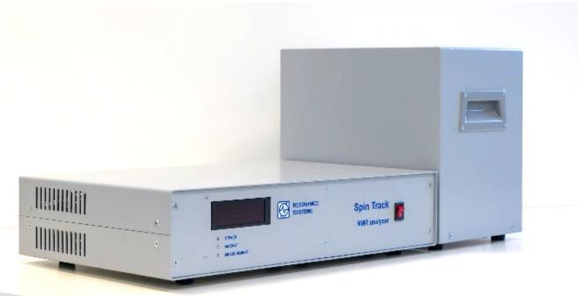

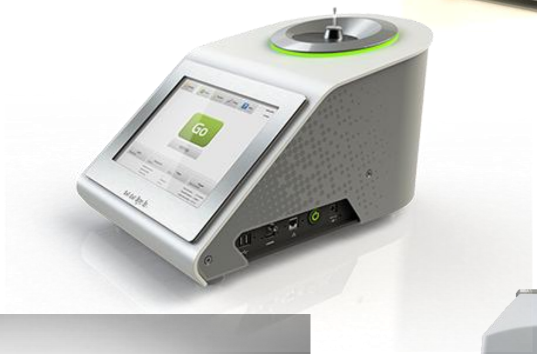

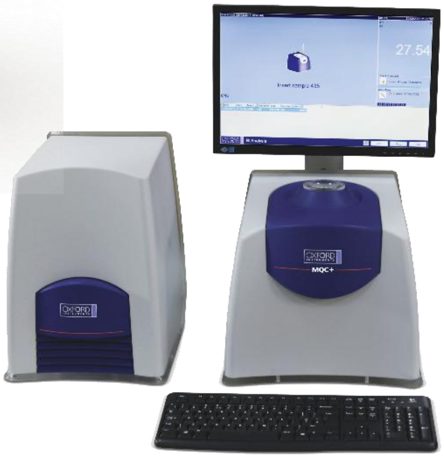

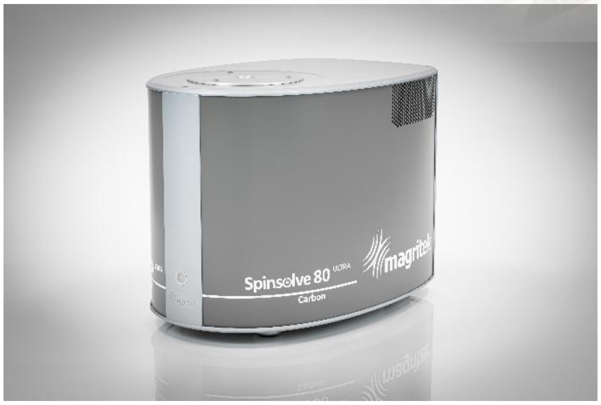

Methods: Time-Domain (TD) vs. Frequency-Domain (FD) Transverse Relaxation Time (T_2) Measurements

Measurements of the transverse relaxation time, T_2, whether in time-domain (TD) or frequency-domain (FD) mode, conventionally employ a Carr-Purcell-Meiboom-Gill (CPMG) pulse sequence.[1,2] Spin dynamics in both modes is the same—creation of transverse magnetization by 90°-pulse, followed by a series of 180°-pulses which alter spin precession and force dephasing/rephasing of spins in the transverse plane. However, in TD mode, one collects the spin-echo signal originating from the dephasing/rephasing process, producing time-dependent echo signal intensity decay data. While in FD mode, the decay of transverse magnetization is monitored, and the resulting free-induction decay (FID) data are converted into an NMR spectrum/signal via Fourier transform (FT).

Schematic presentation of TD measurements of T_2 is shown in Figure SM1.

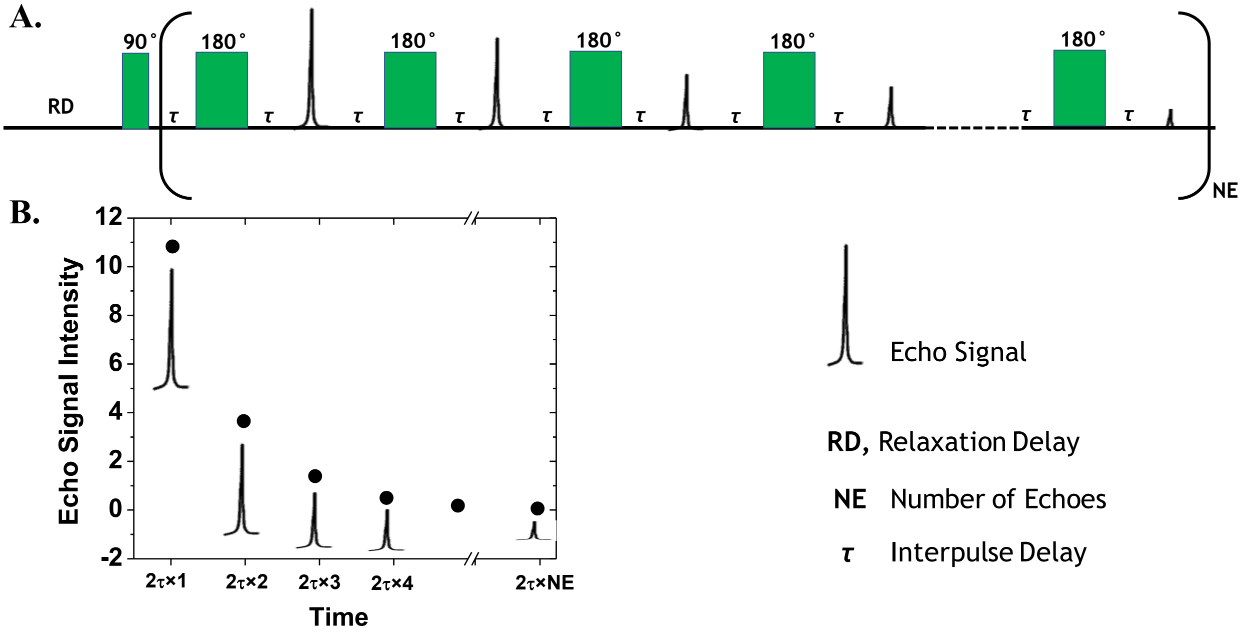

Figure SM1. Time-domain measurements of transverse relaxation time, T_2. (A) Scheme of CPMG pulse train; (B) Model data of decreasing echo signal intensity over time.

In TD mode, after a 90°-pulse, a series of τ-180°-τ cycles is performed with spin-echo signal intensity values collected after each cycle (Figure SM1). The resulting data are represented by the decreasing echo signal intensity over time separated by increments of 2τ. The relaxation delay (RD) as well as the total duration of data collection are both generally selected around 5-fold to 6-fold of T_2. Hence, for the water signal, with a T_2 (at 25 °C) of ca. 2.5 s to 2.7 s, the total duration of a single transient experiment is usually equal to ca. 12 s to 16 s. This time corresponds to the value $2\tau \times NE$, as shown in Figure SM1, and for a conventionally used τ value of (0.5 to 2.0) ms the resulting number of collected echoes, NE, will be in a range from ca. 6,000 to 32,000 echoes. Therefore, in TD mode, a single time-dependent echo signal intensity decay is comprised of

thousands of data points. In most cases, exponential fitting of such a massive data array allows for the extraction of T_2 values of with rather high accuracy.

Schematic presentation of FD measurements of T_2 is shown in Figure SM2.

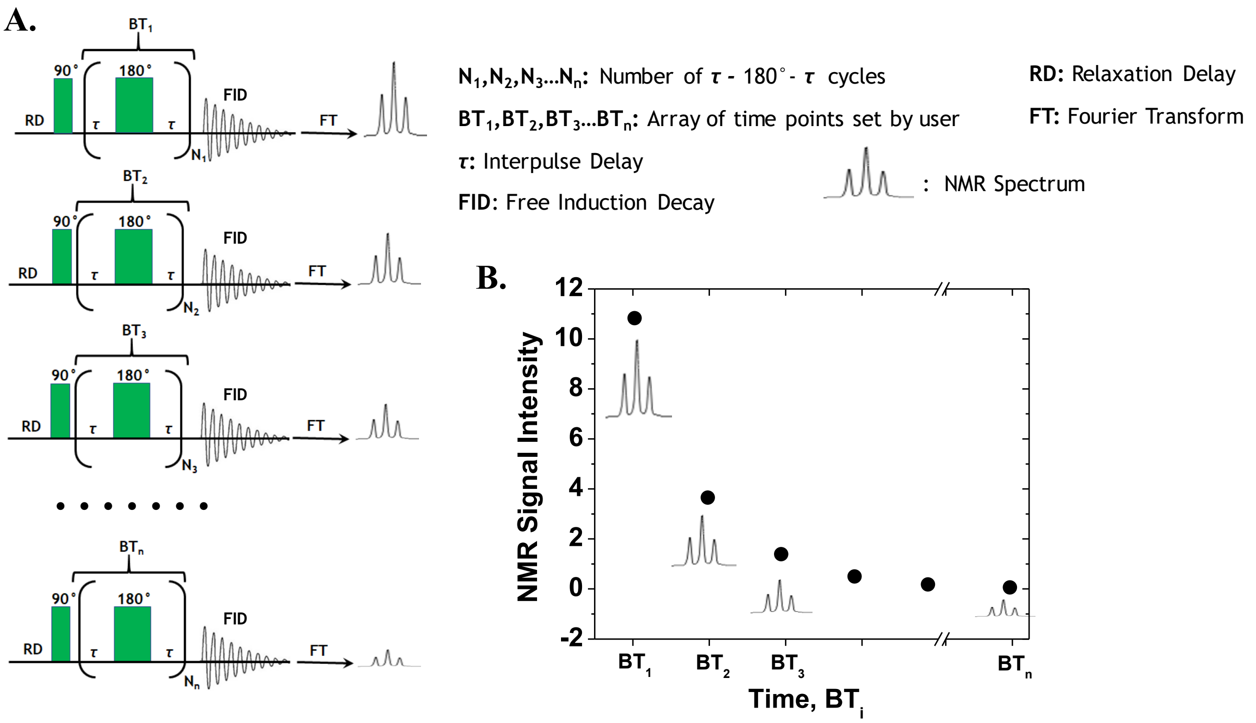

Figure SM2. Frequency-domain measurements of transverse relaxation time, T_2. (A) CPMG pulse scheme; (B) Model relaxation data of NMR signal intensity decay over time.

Unlike TD mode, in which thousands of data points are collected in a single experiment, each data point in FD mode is acquired through a separate experiment. Figure SM2 shows the series of such experiments, each producing a single data point in the plot of NMR signal intensity decay over time. To collect these data points, parameter settings involve creating an array of ascending time values BT_i (oftentimes called "big tau"). In each experiment of such series, the number of τ-180°-τ cycles N_i is performed, where N_i is set to the integer of $BT_i/2\tau$. Next, the resulting FID is detected, and Fourier transformed to obtain the intensity of the NMR signal of interest to plot it vs. corresponding BT_i value. Similar to the TD mode, the collected intensity vs. time decay is fitted with an exponential function to extract the value of T_2. The same as in the TD mode, the relaxation delay (RD) as well as the longest BT_i value are both generally selected around 5-fold to 6-fold that of T_2. Evidently, the number of data points in the FD mode is limited by a feasible length of values in the BT_i array. Therefore, with at least (15 to 20) data points needed for reliable fitting, the measurements of the characteristic T_2 values of water could easily require ca. 1 hour.

Methods: Parameters Selected for CPMG Experiments by Each Lab

Parameters for experiments from each participating Lab.

**Lab#**	**Color**	**^{1}H Frequency (MHz)**	**TD or FD CPMG Pulse Sequence**	**Sample Temp. (°C)**	**NMR tube size (mm)**	**Tau (µs)**	**Expt. Time (min.)**	**Number of Echoes/ Number of points[a]**	**Number of transients/ scans/phase cycling**	**Relaxation Delay (s)[b]**	**Samples were inverted**
Lab1	**Black**	17.9	Time	28	18	250	3 to 8	varies	16	varies	Yes
Lab2	**Red**	19.95	Time	28	18	6000	20	500, mostly	32	n.g.	n.g.
Lab3	**Blue**	23.4	Time	28	18	500	2.5	16,000	8	15	Yes
Lab4	**Green**	60.38	Frequency	33	5	1000	20	16	n.g.	n.g.	Yes
Lab5	**Purple**	19.65	Time	28	18	1000	5.6	1,200 or 1,500	16	18	n.g.
Lab6	**Gold**	80.26	Time	26.5	5	250	1 to 1.2	20,000	4	15	Yes
Lab7	**Aqua**	23.8	Time	25	26	500	1.5 to 2	varies	4	varies	Yes

[a] 'Varies' or where two values are given, indicates that some labs tailored the parameters to each sample based on an initial trial run for each sample.

[b] Information that was not given is listed as n.g.

Methods: Relaxation Data and Analysis

The following describes how the data were treated to fit them to single-exponential models, and to characterize the random and systematic departure of the data from single-exponential behavior.

A total of 267 measurements were submitted from seven labs. Each lab submitted its study data via email or a webform on the study internal webpage hosted by IBBR. The data from each lab was compiled as a single multi-page spreadsheet file, with one measurement per page plus a final page identifying the metadata for each measurement. The study data and associated processing and analysis scripts are available for download in supplementary materials. A given relaxometry measurement was recorded in a page as two columns, specifying time (s) and intensity (arbitrary units), while quadrature NMR measurements were recorded as three columns, specifying time (s), real intensity, and imaginary intensity. Each spreadsheet file was converted to a series of comma separated value (CSV) files, with one file for each page, and these files were converted for input and analysis via the tools of the NMRPipe software system.[3]

For the purposes of the NMRPipe software, measured data from all instruments were treated as complex time domain NMR data, and converted to NMRPipe format for additional analysis. Conversion assumed that the time points are uniformly spaced between the smallest time t_{first} and the longest time t_{max}. If real-only data was submitted, the imaginary part was considered to be all zeros. In the case of data from Lab5, visual inspection showed that the first two points out of ≈ 1,000 points for each curve showed signs of distortion and were omitted from conversion and analysis. A summary of data parameters used by each lab is given in Table ST1, and a representative illustration of data from the seven labs is shown in Figure S1. As shown in Table ST1, there is much variation in measurement parameters between the different labs. The number of points reported ranged from 17 real points (Lab4) to 20,000 complex points (Lab6). The value of t_{max} used varied from 0.128 s (Lab4) to 17.995 s (Lab1), and some labs used the same t_{max} for every measurement (Lab3, Lab5, Lab6), while in other labs, t_{max} was reduced with increasing apparent intensity decay rate (Lab4, Lab7).

Table ST1. Measurement parameters by Lab.

Lab	Data Type	Smallest Size, pts	Largest Size, pts	Smallest t_{first} sec	Largest t_{first} sec	Smallest t_{max} sec	Largest t_{max} sec
**Lab1**	Real	270	589	0.00100	0.06199	0.27000	17.9950
**Lab2**	Real	245	495	0.02422	0.07222	2.01706	6.01706
**Lab3**	Complex	15,982	15,982	0.00300	0.00300	15.9990	15.9990
**Lab4**	Real	17	17	0.00000	0.00000	0.12800	10.0160
**Lab5**	Real	1,198	1,198	0.01229	0.01229	4.90534	4.90534
**Lab6**	Complex	20,000	20,000	0.00050	0.00050	9.99950	9.99950
**Lab7**	Complex	5,000	15,000	0.00100	0.00100	5.00000	15.0000

Single exponential ($S_{obs}(t)$) fitting was performed using the "modelXY" non-linear least squares model fitting application in NMRPipe, with the following model:

$$S_{obs}(t) = A * \exp(-\alpha t) + b \quad \text{[Eq. S1]}$$

where A is the signal amplitude, α is the decay rate (s^{-1}), t is time (s), and b is an offset correction. As shown in Figure S1, data from some labs can be seen to decay to zero (Lab3, Lab7), while some labs have a visible offset, in particular Lab1, where signals decay to negative values.

Given the nature of the samples, it is expected that data from all labs predominately consists of single-exponential signals, and so the residual of a single exponential fit should reveal whether a given sample is exhibiting more complex relaxation behavior. However, in order to discern this, it is necessary to identify other sources of variation in the data such as random thermal noise and systematic measurement artifacts. Regarding random noise level, this can be difficult to estimate directly from the time-domain data, unless the measurement is extended until the signal has fully decayed. While it would seem reasonable to estimate the noise from the tail end of a fully decayed relaxation signal, Fourier analysis will show that systematic artifacts can make this estimate misleading (Figure S2).

Fourier Transform Used to Reveal Systematic Distortion

While relaxometry data is usually analyzed in the time-domain, Fourier transforming the measured data reveals important details about signal and artifacts (Figure S2). These spectra were prepared by treating the relaxometry data as a complex time-domain measurement with the imaginary part set to zero if imaginary data was not reported. Before complex Fourier transform, this time domain data was offset corrected by subtracting the average of the last 10 % of time points, and zero filled to the smallest power of two that is greater than or equal to twice the original data size. The Fourier transform of an "ideal" single exponential relaxometry signal should give an approximate Lorentzian line shape centered at zero frequency, the shape being approximate due to the nature of discrete Fourier processing. As noted above, it is expected that the measurement will contain random thermal noise, which would show up in the spectrum as a uniform random noise baseline, and therefore baseline segments of the spectrum can be used to deduce the corresponding random noise level in the original time-domain data. As shown in Figure S2, the Fourier transform also reveals that several measurements have non-decaying RF oscillation artifacts such as from alternating current (AC) power sources or transformer units (several hundred Hz), which show up as spikes in the spectrum, with spike artifacts especially prominent in data from Lab5 and Lab7. This is critical knowledge, because it shows that even if a relaxometry signal decays completely, the tail end of the relaxation curve is not always a good indicator of random noise level, because it potentially consists of both random noise and the non-decaying oscillation artifacts.

Artifacts Due to Eddy Currents

In addition to random thermal noise, it is expected that there might be systematic artifacts including decaying RF oscillations from the instrument and from the nonmagnetic but conductive metal vial cap, and these are revealed by inspection of residuals from single exponential fit. An example of these artifacts is shown in Figure S3. In most cases except for Ferrlecit® samples, these artifacts

are small, and well-described by a single rapidly decaying signal of arbitrary phase. Based on visual inspection, only data from Lab1, Lab3, and Lab6 were analyzed by including this decaying oscillation term in the model. This correction procedure is not ideal for samples that have multi-exponential behavior, because the decaying oscillation term can adopt a frequency close to zero Hz so that it corresponds to a simple exponential. The impact of sinusoidal correction had only a small effect on the $R_2(^1H_2O)$, changing the $R_2(^1H_2O)$ by a root mean square (RMS) of ~3.6 % in this representative plot of data before and after correction for eddy currents (Figure S4).

Phase Imperfections in Complex Measurements

Some data from Lab3, Lab6, and Lab7 are conventional complex time-domain measurements, which would ideally have an on-resonance signal of zero phase, so that the imaginary part is zero. In practice, small non-zero imaginary signals are seen (Figure S5). To adjust for this, complex data were automatically phase-corrected by grid search in 0.01 degree steps to minimize the RMS of the first 15 % of imaginary points. The impact of this phase correction on the $R_2(^1H_2O)$ was very small as shown in Figure S6, changing the $R_2(^1H_2O)$ values before and after phase correction only by a RMS of ~0.0006 %.

Analysis of the Contributions to the Signal

Given the above considerations, to characterize the decay rates and estimate errors, we will describe the measured signal $\mathbf{S_{total}}$ in terms of the underlying exponentially decaying signal S_{obs} and additional perturbations:

$$\mathbf{S_{total}} = \mathbf{S_{obs}} + \mathbf{S_{other}} + \mathbf{S_{eddy}} + \mathbf{S_{noise}} + \mathbf{S_{ac}} + \mathbf{S_{ps}} \qquad \text{[Eq. S2]}$$

where $\mathbf{S_{other}}$ is any additional multiexponential decay signal from the sample, $\mathbf{S_{eddy}}$ describes the rapidly decaying oscillation artifacts due to eddy currents etc., $\mathbf{S_{noise}}$ is the random thermal noise, $\mathbf{S_{ac}}$ are the non-decaying artifacts due to AC power etc., and $\mathbf{S_{ps}}$ is the discrepancy due to imperfectly phased complex data, which as noted above can be derived from the early part of the imaginary data. The value for $\mathbf{S_{noise}}$ can be derived from a random noise estimate taken from the spectrum and back calculated to the time-domain. If the data is dominated by a single exponential signal, as expected in this study, the early part of a single-exponential residual would show the presence of both any "true" multi-exponential behavior $\mathbf{S_{other}}$, and also the decaying oscillation artifacts $\mathbf{S_{eddy}}$. Likewise, the tail part of a single exponential residual would show both the random noise S_{noise} and non-decaying artifacts $\mathbf{S_{ac}}$, so that knowledge of $\mathbf{S_{noise}}$ from the spectrum allows estimate of $\mathbf{S_{ac}}$ in the time-domain. It should be noted that there are other measurement details that can contribute to error besides the perturbations accounted for here, including incorrectly set relaxation delays.

The goals of analysis are to extract the exponential decay rate from term $\mathbf{S_{obs}}$, gauge the uncertainty in this estimate due to $\mathbf{S_{eddy}}$, $\mathbf{S_{noise}}$, $\mathbf{S_{ac}}$ and $\mathbf{S_{ps}}$, and optionally to decide whether the data justifies modeling of additional multiexponential behavior $\mathbf{S_{other}}$. When $\mathbf{S_{noise}}$, $\mathbf{S_{ac}}$, and $\mathbf{S_{ps}}$ are all known and accounted for, the remaining residual in a single exponential fit can be attributed to $\mathbf{S_{eddy}}$ + $\mathbf{S_{other}}$. While these terms must be separated by non-linear model fitting, values for $\mathbf{S_{noise}}$, $\mathbf{S_{ac}}$, and $\mathbf{S_{ps}}$ do not require model fitting, so that it is possible to place an upper limit on the size of any true

multiexponential behavior $\mathbf{S_{other}}$, according to the magnitude of the single exponential residual after random noise and AC noise is accounted for. Estimated signal contributions for Lab1, Lab3, and Lab6, which included eddy current model terms, are shown in Figure S7. Estimates of signal contributions for Lab2, Lab5, and Lab7, were fit only to single exponentials (Figure S8). Residual analysis for Lab4, which does not have detailed analysis of signal terms because the data curves include only 17 points, is shown in Figure S9.

Estimation of Error in Single Exponential Model Decay Parameter

Several approaches can be used to estimate the errors in the extracted exponential decays. To investigate the impact of random variation, each exponential fit was repeated 20 times using a randomly selected subset including 90 % of the measured points. An example is shown in Figure S10. Likewise, in order to estimate the impact of systematic variation, such as S_{eddy} which effects early time points more than later ones, the exponential fit was also repeated by systematically omitting a 10 % range of points, and moving this range by 5 % for each fit, so that one fit was done by omitting points from 0 % to 10 %, the next fit by omitting points from 5 % to 15 %, then 10 % to 20 %, etc. An example is shown in Figure S11. As shown in Figures S10 and S11, the systematic variations are larger than the variations due to random noise, so these larger values were used as the error estimates for the alpha decay rate.

Given knowledge of the random and systematic variations, it would also be possible to perform a more detailed Monte Carlo error analysis, where an ideal exponential is first simulated, then perturbed artificially, according to typical patterns of random and systematic noise, and finally an exponential decay is recovered by fitting. However, since the simple estimates of uncertainty in the exponential decays are shown to be so much lower than operational variation, this error analysis was not performed.

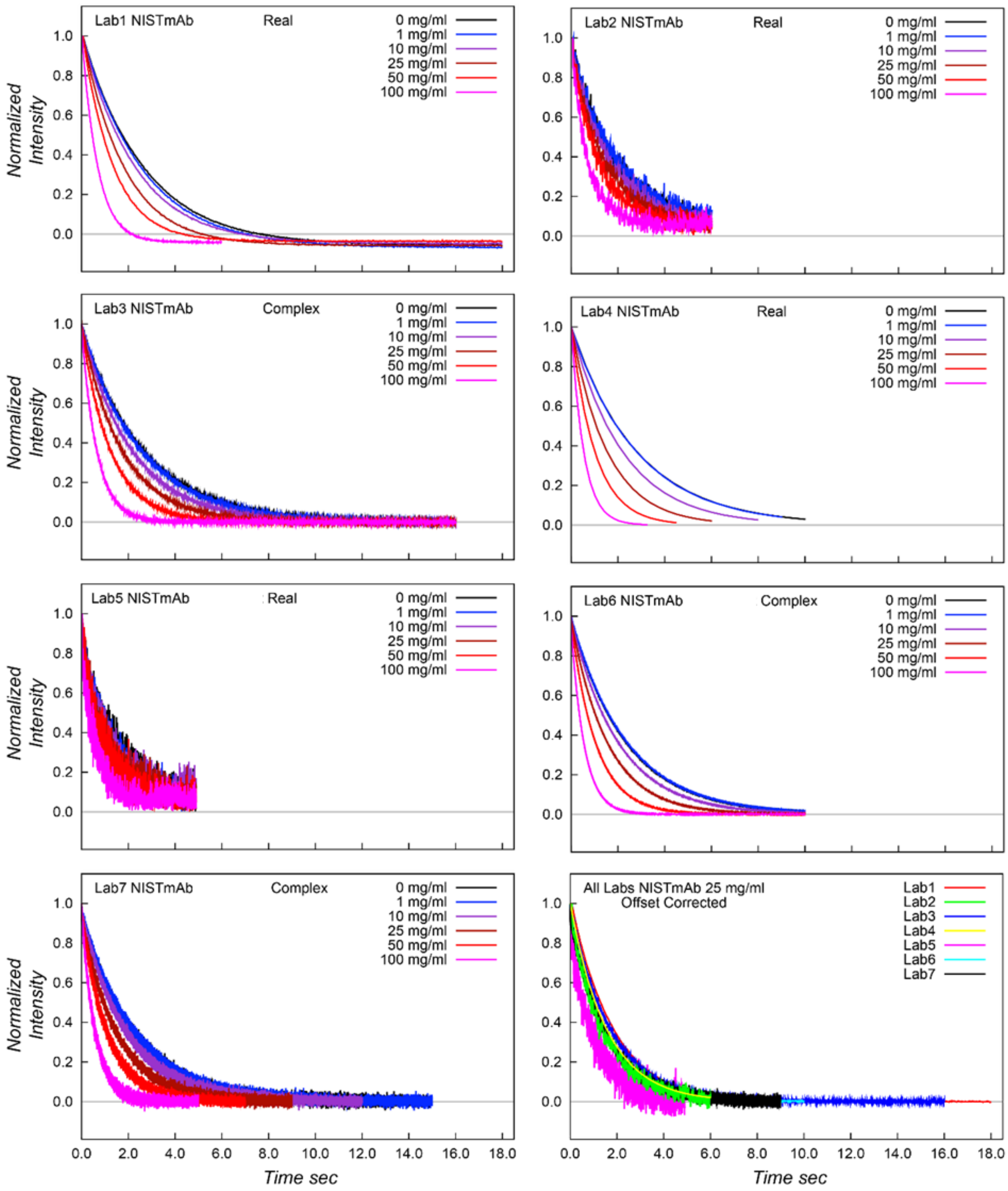

Figure S1. Representative data from each lab, with maximum values for each curve scaled to 1, and all data displayed over the same time range. As shown, some labs, for example Lab4 and Lab7, used different t_{max} values for each sample. The curves displayed for "All Labs" in the lower right have been offset-corrected by fitting a single exponential plus a constant and then subtracting the constant from the original data.

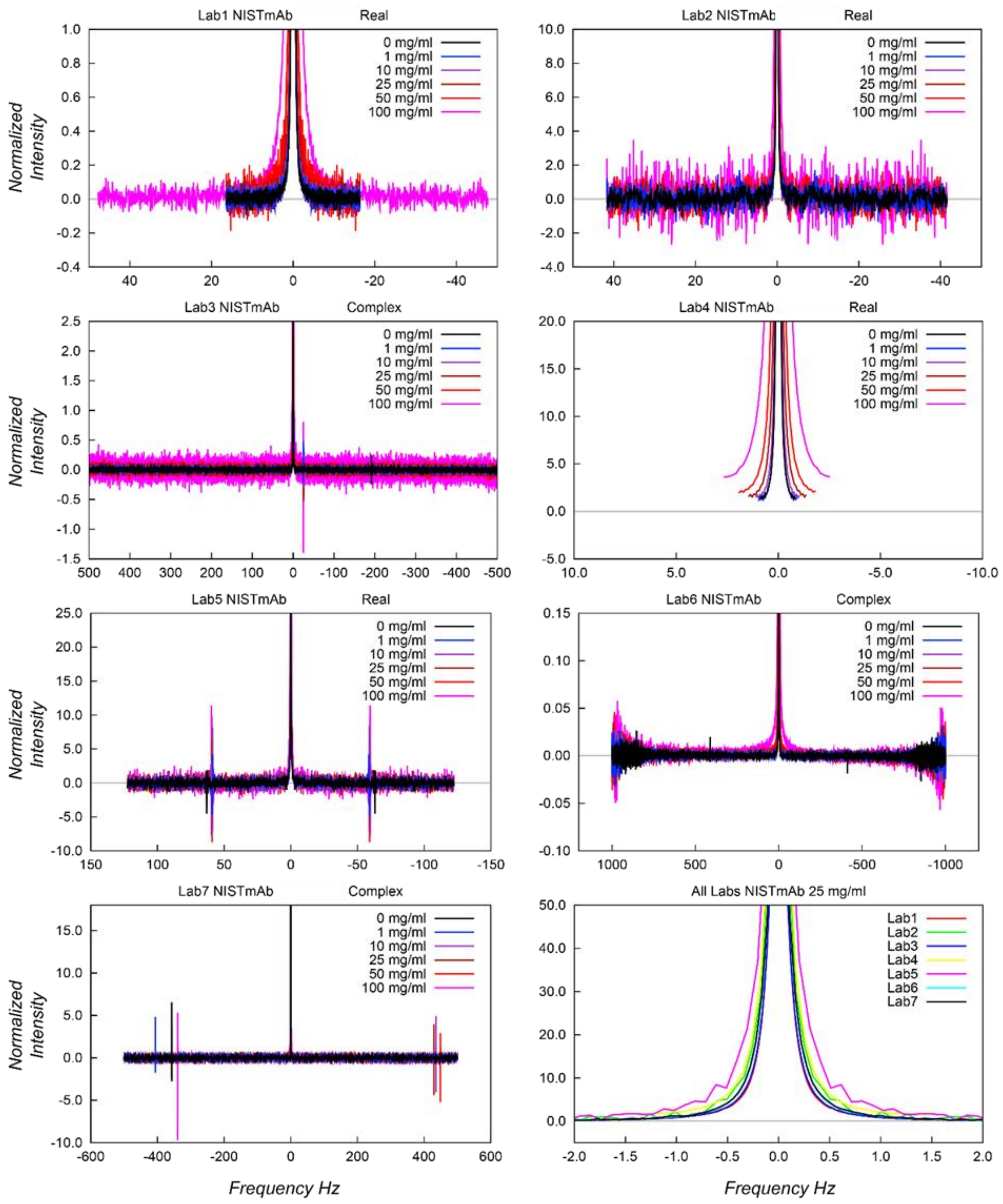

Figure S2. Fourier transform of representative relaxation data for each lab, with maximum values for each spectrum scaled to the spectral width in Hz of the 100 mg/mL sample. Spectral processing details are described in the text. In the Fourier transform representation, the width of the zero-frequency peak is a measure of the relaxation rate. As noted in Figure S1, some labs used different t_{max} values for different samples spectral widths differed as well. The spectra displayed for "All Labs" in the lower right shows an expanded region with a spectral range common to all measurements. In several cases, such as shown for Lab5, AC power artifacts that cause a small oscillating signal in the relaxation measurement are revealed as spikes in the Fourier transform. Since these signals are small compared to the thermal noise, they are not readily identifiable in the raw relaxation data. In addition, since they are constant and much faster than the relaxation decay, they have the same effect on exponential fitting as random thermal noise.

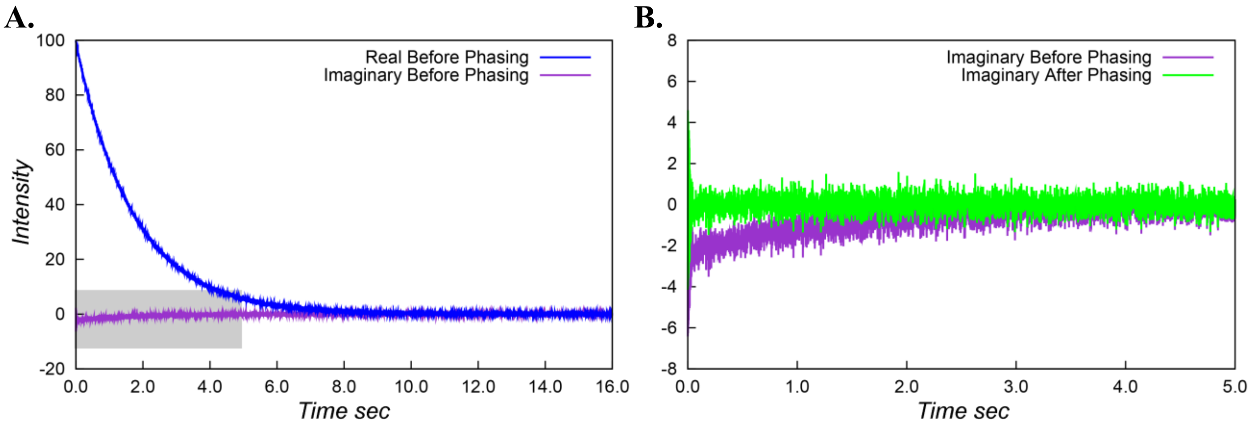

Figure S3. Example phase correction of phase imperfection, as shown in the Lab3 NISTmAb 25 mg/ml sample. (A) real and imaginary parts of the measured data before phase correction. In a frequency-domain relaxation measurement, only the real part of the data is analyzed, and if the data are perfectly phased, the imaginary component should be flat, and all the information of interest is contained in the real part of the data. (B) expanded region showing imaginary data before and after automated phase correction. A phase correction maintains the magnitude of the data. When the imaginary data is increased by this small amount, the corresponding real data is reduced by a complementary small amount. The automated procedure finds the phase which minimizes the RMS of the first 15 % of imaginary points, using a grid search in 0.01 degree steps.

A. **B.**

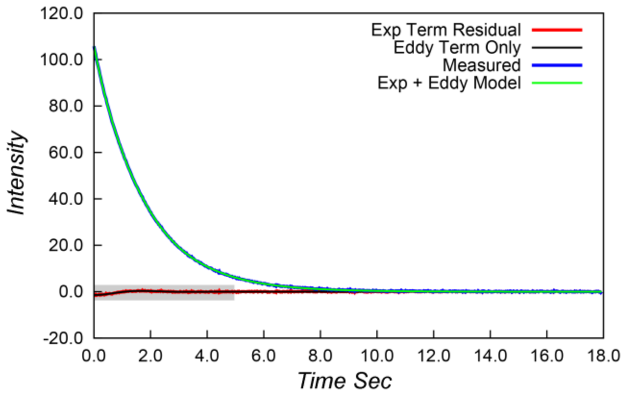

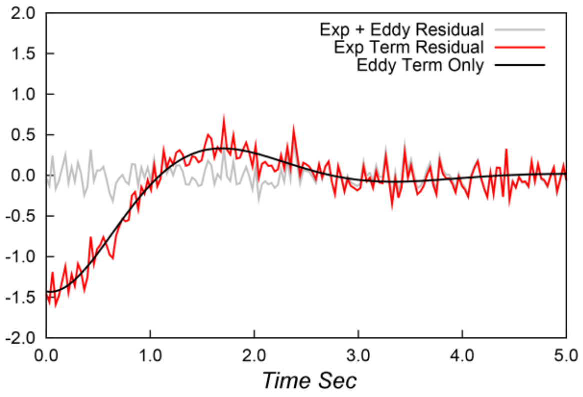

Figure S4. Example data demonstrating eddy current distortion and correction, as shown for the Lab1 NISTmAb 25 mg/ml sample. This data is modeled as an exponential term plus an eddy term, which is a decaying sinusoid. (A) measured data and model terms. (B) expanded view, showing the eddy distortion, the eddy model term, and the final residual. In most cases, the amplitude of the eddy adjustment is less than 5 % of maximum intensity, as shown here.

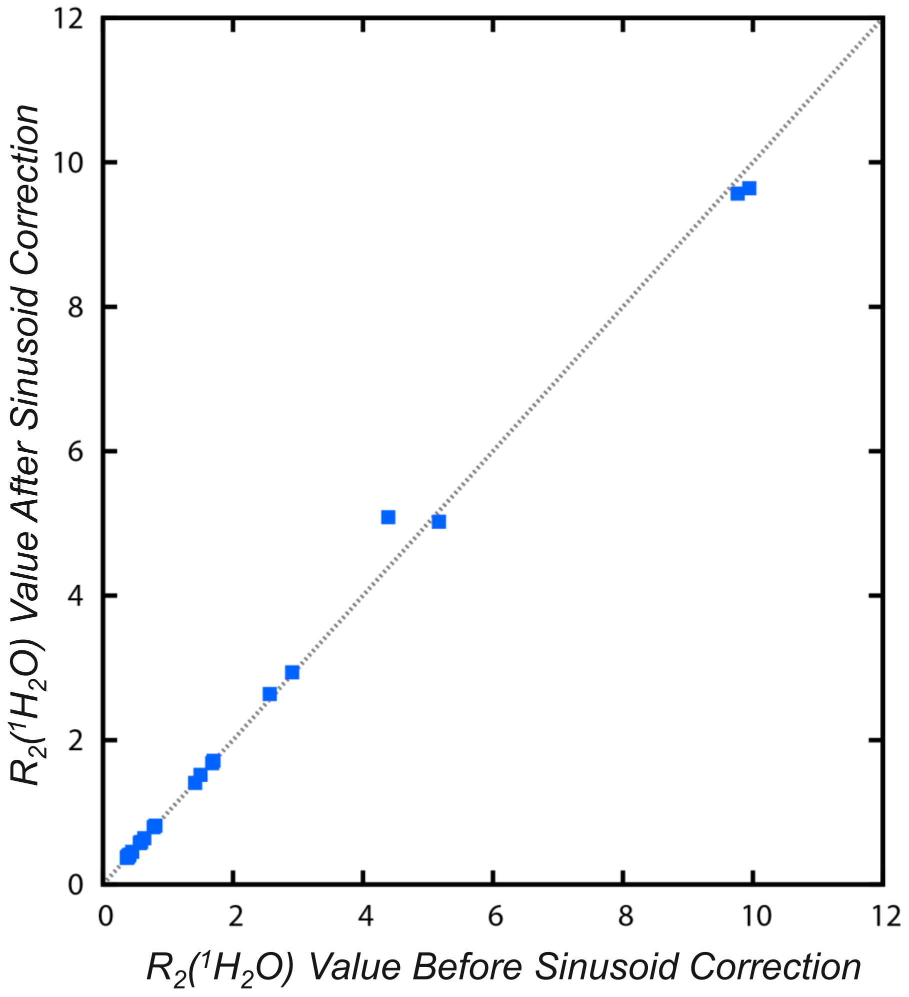

Figure S5. Representative $R_2(^1H_2O)$ data are plotted before and after sinusoid correction was applied. The sinusoid correction has a small effect on the $R_2(^1H_2O)$ values, which changed by RMS ~3.6 %, and become systematically worse for samples with faster decays.

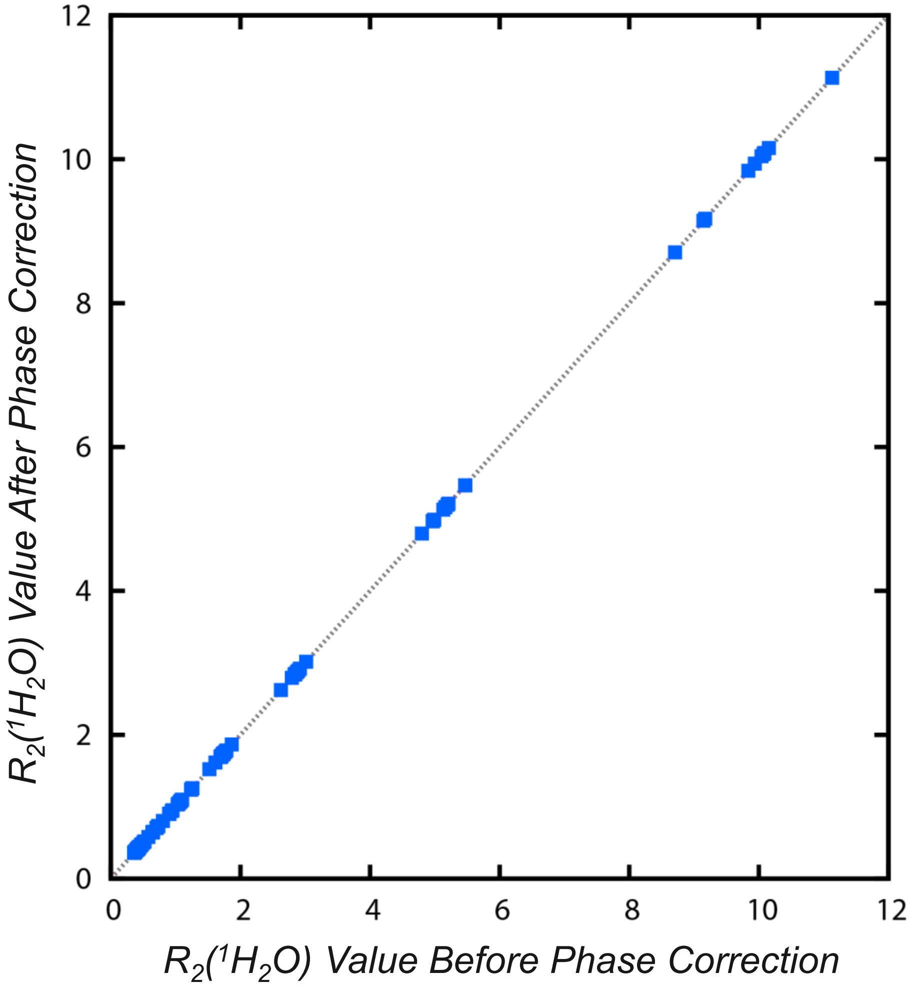

Figure S6. Representative data show the effect of phase correction on the extracted $R_2(^1H_2O)$ values. The values change by a root mean square (RMS) of ~0.0006 %. The small difference between the $R_2(^1H_2O)$ values before and after phase correction indicates the data phasing does not significantly contribute to the $R_2(^1H_2O)$. Automatic phase correction was applied to the imaginary component of all complex data.

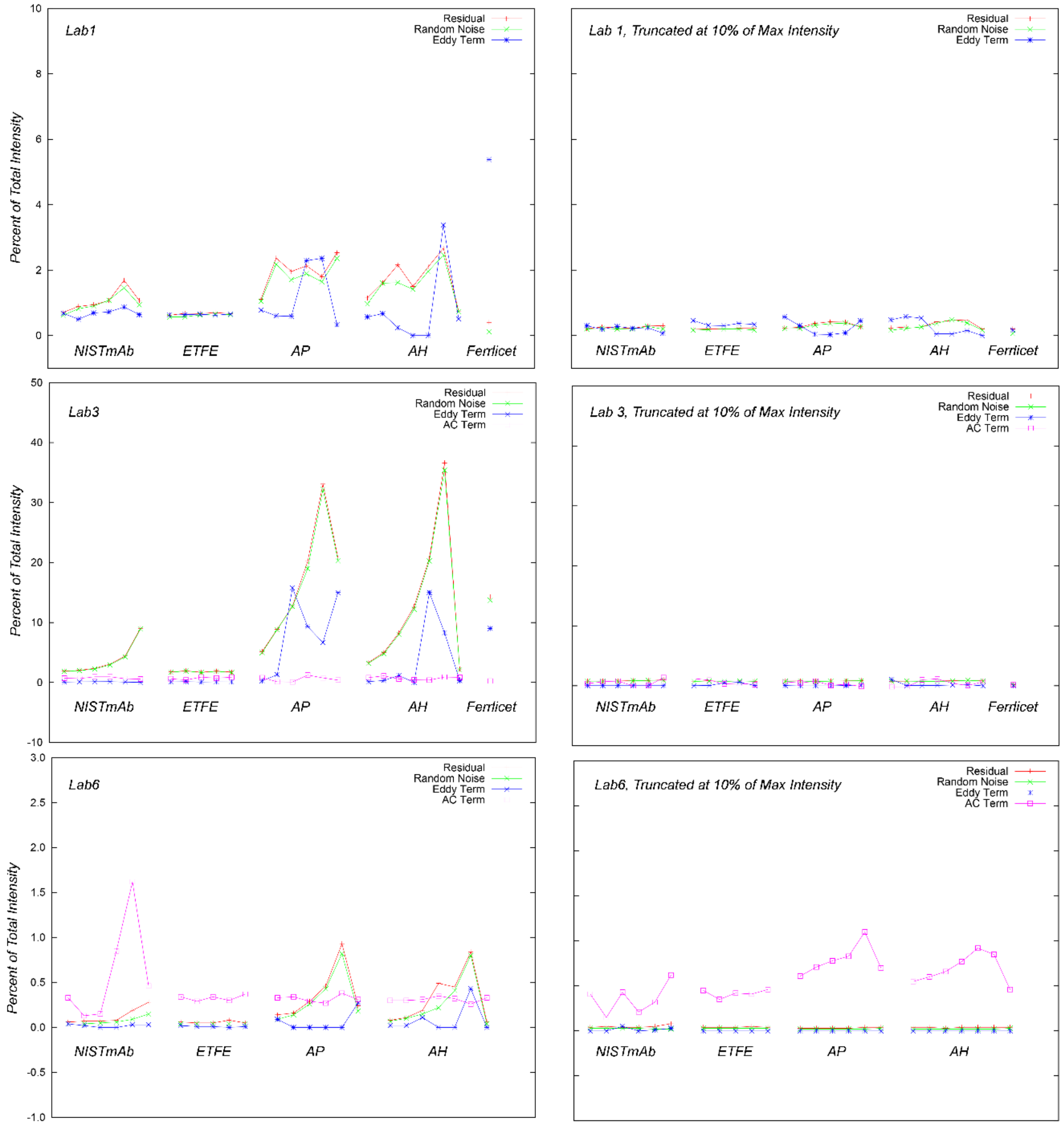

Figure S7. Estimated signal contributions for Lab1, Lab3, and Lab6, which were modeled with an eddy current term. This model is not ideal for samples that have multi-exponential behavior, because the decaying oscillation term can adopt a frequency close to zero Hz so that it corresponds to a simple exponential. For this reason, any eddy current term which was greater than two times larger than the original single exponential residual was rejected. Because of the wide range of acquisition parameters used, noise statistics for the full measurements are not directly comparable between labs, especially in cases with larger numbers of points and larger t_{max}. For clearer inter-lab comparison, the left side panels show statistics for all points in the full measurements, and the right-side panels show the same statistics for data with size truncated at the first point that falls below 10 % max intensity.

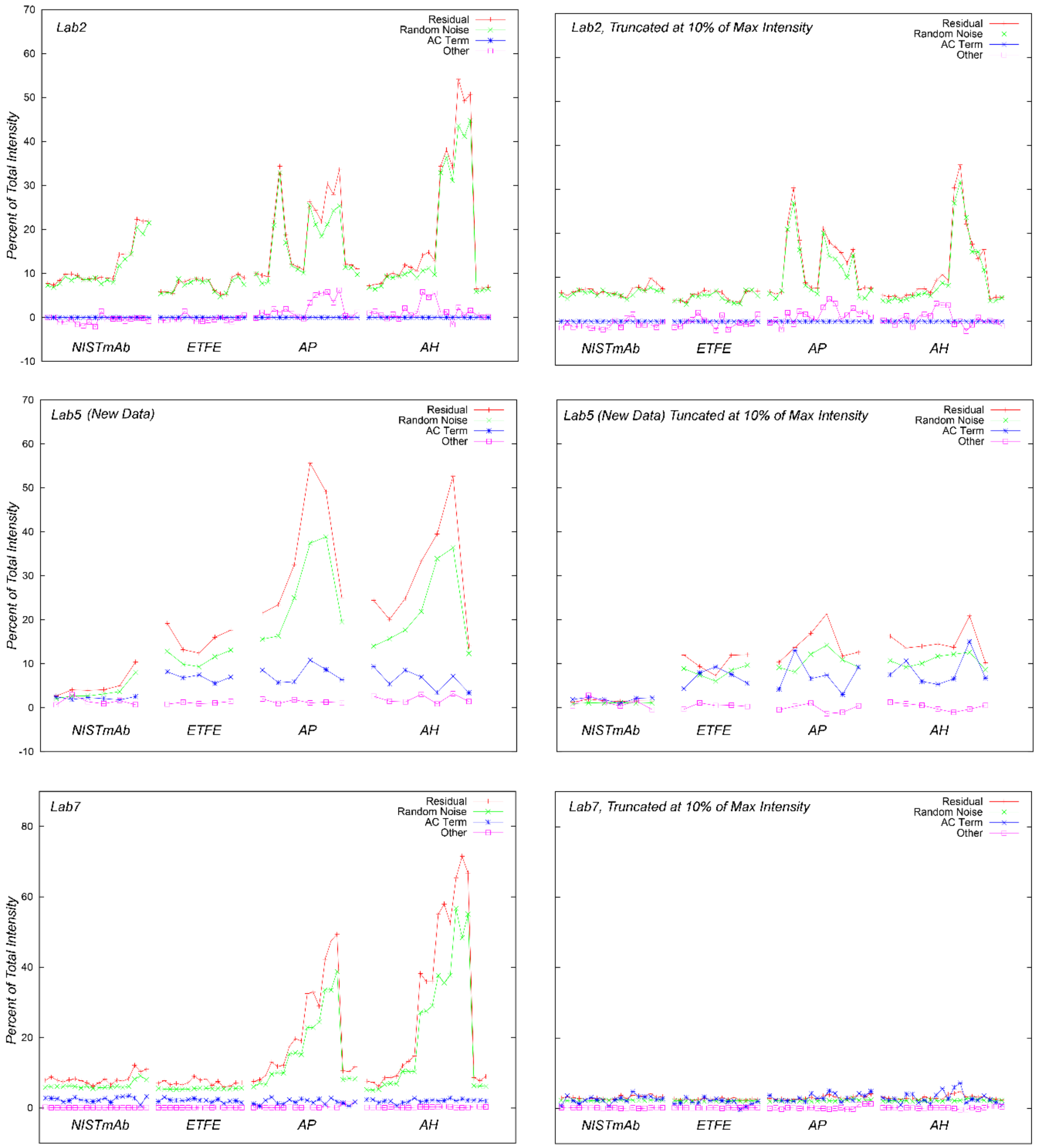

Figure S8. Estimated signal contributions for Lab2, Lab5, and Lab7. As in Fig. S7, for clearer inter-lab comparison, the left panels show statistics for all points in the full measurements, and the right panels show the same statistics for data with size truncated at the first point that falls below 10 % max intensity.

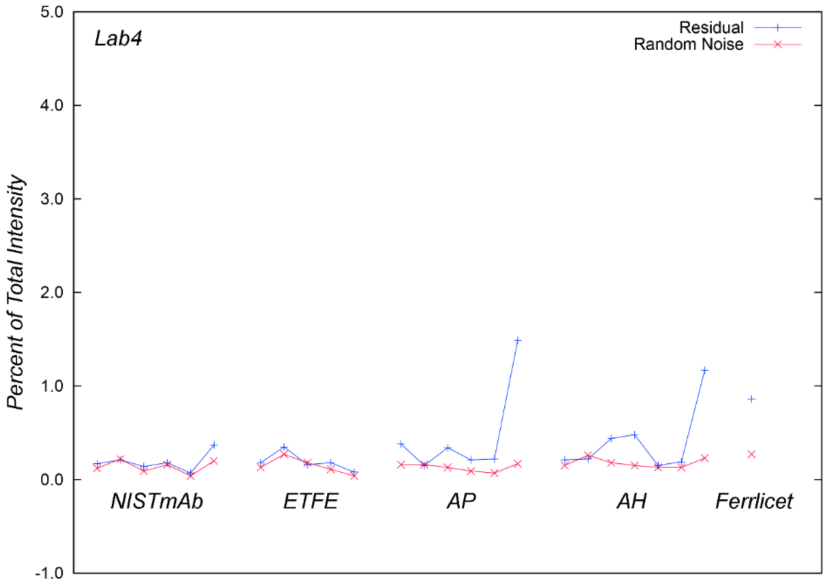

Figure S9. Estimated signal contributions for Lab4. Detailed analysis of signal components was not performed on this data, which only contains 17 points per curve.

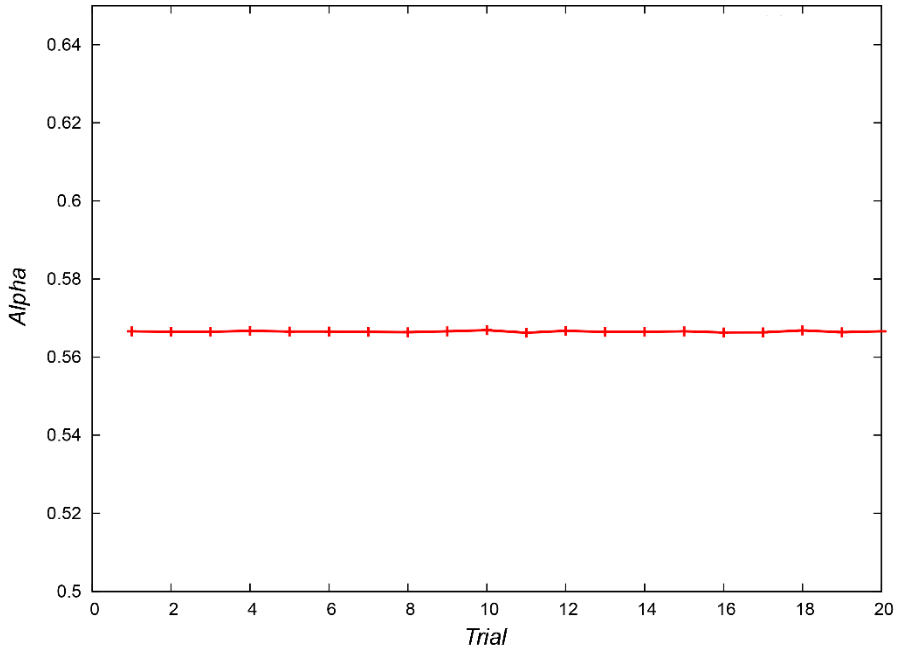

Figure S10. Example alpha decay rate parameter with random point deletion. Multiple trials were performed with random deletion of 10 % of the points. Data for Lab1 NISTmAb 25 mg/ml sample is shown.

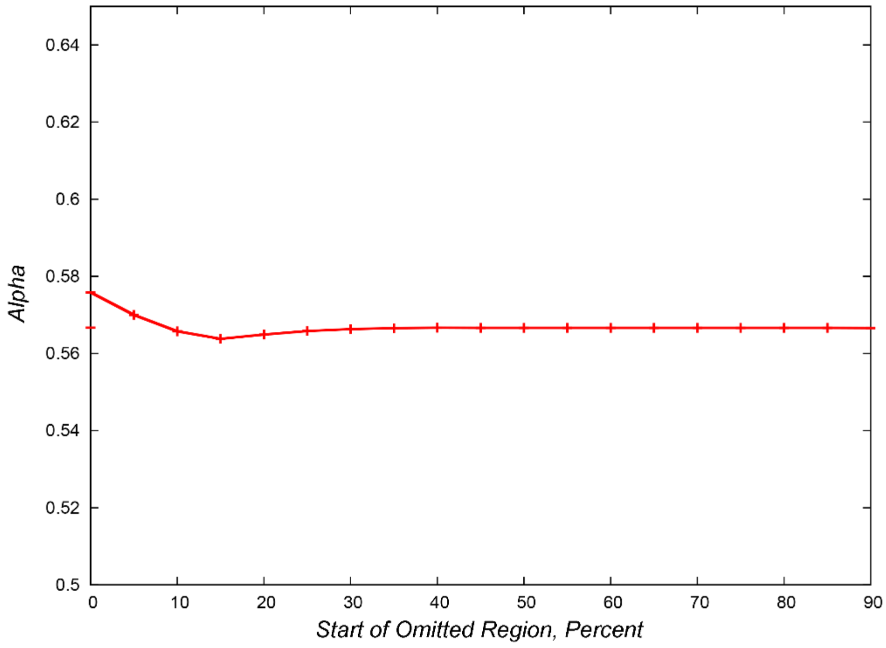

Figure S11. Example alpha decay rate parameter extracted with systematic point deletion. Multiple trials were performed, with systematic deletion of a 10 %-wide range of points. Data for Lab1 NISTmAb 25 mg/ml sample is shown.

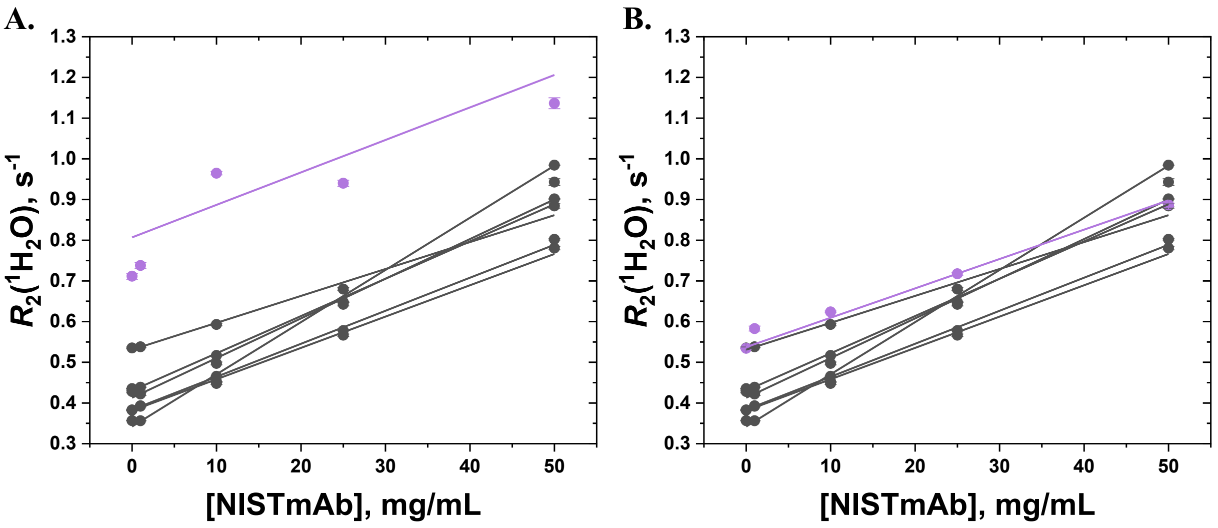

Figure S12. Relaxivity of NISTmAb before and after optimizing the detection angle. NISTmAb concentrations were from 0 to 50 mg/mL with measurements from one outlier lab (purple) (A) before and (B) after adjusting the sample height, which improved the signal to noise ratio. Operational variability was the largest contributor to data variability.

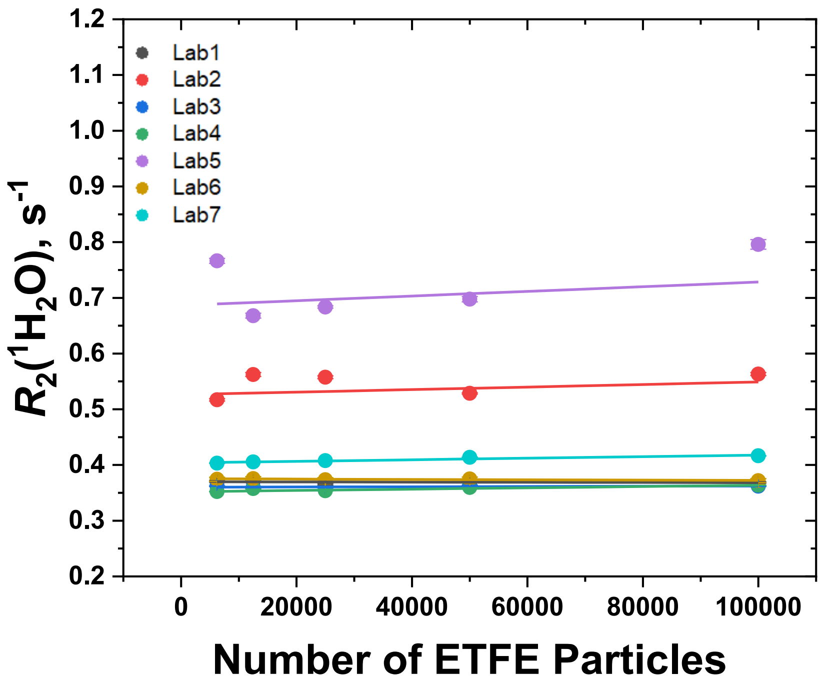

Figure S13. $R_2(^1H_2O)$ of NIST ETFE particles (RM8634, 6,250 to 100,000 particles).

Table ST2. Relaxivities of NISTmAb.

Lab Code	$R_2(^1H_2O)$ relaxivity, $(mg/mL)^{-1}s^{-1}$	$<R^2>$
Lab1	$(7.70 \pm 0.29) \times 10^{-3}$	0.996
Lab2	$(6.61 \pm 0.38) \times 10^{-3}$	0.990
Lab3	$(8.11 \pm 0.19) \times 10^{-3}$	0.998
Lab4	$(12.82 \pm 0.24) \times 10^{-3}$	0.999
Lab5	$(7.21 \pm 0.43) \times 10^{-3}$	0.989
Lab6	$(9.75 \pm 0.68) \times 10^{-3}$	0.986
Lab7	$(9.16 \pm 0.26) \times 10^{-3}$	0.998

Table ST3. Relaxivities of NIST ETFE particles.

Lab Code	$R_2(^1H_2O)$ relaxivity, $(mg/mL)^{-1}s^{-1}$	$<R^2>$
Lab1	$(-1.64 \pm 2.85) \times 10^{-8}$	0.100
Lab2	$(0.23 \pm 0.33) \times 10^{-8}$	0.134
Lab3	$(2.26 \pm 2.72) \times 10^{-8}$	0.188
Lab4	$(0.12 \pm 1.25) \times 10^{-8}$	0.967
Lab5	$(0.42 \pm 0.01) \times 10^{-8}$	0.049
Lab6	$(-2.89 \pm 1.43) \times 10^{-8}$	0.577
Lab7	$(0.14 \pm 2.46) \times 10^{-8}$	0.913

Table ST4. Relaxivities of Alhydrogel®.

Lab Code	$R_2(^1H_2O)$ relaxivity, $(mg/mL)^{-1}s^{-1}$	$<R^2>$	RMSD
Lab1	0.89 ± 0.06	0.984	0.35
Lab2	0.24 ± 0.06	0.786	0.33
Lab3	1.08 ± 0.00	0.999	0.13
Lab4	1.08 ± 0.03	0.998	0.10
Lab5	0.33 ± 0.03	0.975	0.21
Lab6	0.92 ± 0.03	0.997	0.11
Lab7	1.07 ± 0.02	0.999	0.52

Table ST5. Relaxivities of Adju-Phos®.

Lab Code	$R_2(^1H_2O)$ relaxivity, $(mg/mL)^{-1}s^{-1}$	$<R^2>$	RMSD
Lab1	2.12 ± 0.20	0.975	0.46
Lab2	-0.04 ± 0.12	0.034	1.39
Lab3	1.89 ± 0.02	0.999	0.08
Lab4	2.17 ± 0.07	0.997	0.10
Lab5	0.90 ± 0.24	0.828	0.32
Lab6	1.82 ± 0.08	0.995	0.28
Lab7	1.91 ± 0.11	0.991	0.46

Table ST6. Detection of freeze/thaw damaged Alhydrogel® sample.

Lab Code	$R_2(^1H_2O)$ frozen/ $R_2(^1H_2O)$ unfrozen	% Change
Lab1	0.29	71
Lab2	0.70	30
Lab3	0.25	75
Lab4	0.25	75
Lab5	0.58	42
Lab6	0.25	75
Lab7	0.27	73

Table ST7. Detection of freeze/thaw damaged Adju-Phos® sample.

Lab Code	$R_2(^1H_2O)$ frozen/ $R_2(^1H_2O)$ unfrozen	% Change
Lab1	0.58	42
Lab2	1.44	44
Lab3	0.59	41
Lab4	0.61	39
Lab5	0.90	10
Lab6	0.65	35
Lab7	0.44	56

Table ST8. Reported relaxation rates of Ferrlecit® by four out of seven labs, measured at different temperatures, τ values, and field strengths, listed by lab.

**Lab No.**	**$R_2(^1H_2O)$, s^{-1}**
Lab1	21.626 ± 0.252
Lab3	20.348 ± 0.279
Lab4	38.116 ± 0.150
Lab7	21.816 ± 0.190

Table ST9. Variables amongst benchtop NMR instruments identified in this interlaboratory study.

Hardware & Calibration	CPMG Pulse Sequence Parameters	Sample Handling
B_0 and B_1	Frequency- or Time- domain	Mixing/inverting samples
Probe (sample height, bore size, temp., detection angle)	Inter-pulse delay, τ, Echo time, pulse separation	Temperature equilibration
Tune ^{1}H frequency, resonance offset (O1)	Relaxation Delay/Recycle delay/inter-scan delay/repetition time/repetition delay	Sealed vial or 5 mm NMR tube
Tune 90° & 180° pulse lengths/widths	Number of echoes	
Tune detection angle	Number of transients/scans/cycling	
	Pulse phase cycling (+y, -y, …)	
	Measurement/experiment time	

References

(1) Meiboom, S.; Gill, D. Modified Spin-Echo Method for Measuring Nuclear Relaxation Times. *Rev. Sci. Instrum.* **1958**, *29* (8), 688–691. https://doi.org/10.1063/1.1716296.

(2) Carr, H. Y.; Purcell, E. M. Effects of Diffusion on Free Precession in Nuclear Magnetic Resonance Experiments. *Phys. Rev.* **1954**, *94* (3), 630–638. https://doi.org/10.1103/PhysRev.94.630.

(3) Delaglio, F.; Grzesiek, S.; Vuister, G. W.; Zhu, G.; Pfeifer, J.; Bax, A. NMRPipe: A Multidimensional Spectral Processing System Based on UNIX Pipes. *J. Biomol. NMR* **1995**, *6* (3), 277–293. https://doi.org/10.1007/BF00197809.
